# Landscape of molecular crosstalk between SARS-CoV-2 infection and cardiovascular diseases: emphasis on mitochondrial dysfunction and immune-inflammation

**DOI:** 10.1186/s12967-023-04787-z

**Published:** 2023-12-16

**Authors:** Shiyu Dai, Ting Cao, Han Shen, Xuejing Zong, Wenyu Gu, Hanghang Li, Lei Wei, Haoyue Huang, Yunsheng Yu, Yihuan Chen, Wenxue Ye, Fei Hua, Hongyou Fan, Zhenya Shen

**Affiliations:** Department of Cardiovascular Surgery of the First Affiliated Hospital and Institute for Cardiovascular Science, Suzhou Medical College, Soochow University, Suzhou, 215006 China

**Keywords:** SARS-CoV-2, COVID-19, Cardiovascular diseases, Mitochondria, Immune, Inflammation

## Abstract

**Background:**

SARS-CoV-2, the pathogen of COVID-19, is a worldwide threat to human health and causes a long-term burden on the cardiovascular system. Individuals with pre-existing cardiovascular diseases are at higher risk for SARS-CoV-2 infection and tend to have a worse prognosis. However, the relevance and pathogenic mechanisms between COVID-19 and cardiovascular diseases are not yet completely comprehended.

**Methods:**

Common differentially expressed genes (DEGs) were obtained in datasets of human induced pluripotent stem cell-derived cardiomyocytes (hiPSC-CMs) infected with SARS-CoV-2 and myocardial tissues from heart failure patients. Further GO and KEGG pathway analysis, protein–protein interaction (PPI) network construction, hub genes identification, immune microenvironment analysis, and drug candidate predication were performed. Then, an isoproterenol-stimulated myocardial hypertrophy cell model and a transverse aortic constriction-induced mouse heart failure model were employed to validate the expression of hub genes.

**Results:**

A total of 315 up-regulated and 78 down-regulated common DEGs were identified. Functional enrichment analysis revealed mitochondrial metabolic disorders and extensive immune inflammation as the most prominent shared features of COVID-19 and cardiovascular diseases. Then, hub DEGs, as well as hub immune-related and mitochondria-related DEGs, were screened. Additionally, nine potential therapeutic agents for COVID-19-related cardiovascular diseases were proposed. Furthermore, the expression patterns of most of the hub genes related to cardiovascular diseases in the validation dataset along with cellular and mouse myocardial damage models, were consistent with the findings of bioinformatics analysis.

**Conclusions:**

The study unveiled the molecular networks and signaling pathways connecting COVID-19 and cardiovascular diseases, which may provide novel targets for intervention of COVID-19-related cardiovascular diseases.

**Supplementary Information:**

The online version contains supplementary material available at 10.1186/s12967-023-04787-z.

## Background

The coronavirus disease 2019 (COVID-19), caused by the severe acute respiratory syndrome-coronavirus 2 (SARS-CoV-2) infection, has resulted in a global pandemic. In hospitalized COVID-19 patients, acute clinical cardiovascular manifestations are prevalent, including myocarditis, arrhythmias, myocardial ischemia, cardiogenic shock, and acute heart failure [1]. Furthermore, at least 10% of COVID-19 survivors develop persistent symptoms for more than three months following their initial infection, a condition recognized as “long COVID”. Cardiovascular indications, such as hypotonia, palpitations, tachycardia, and chest pain, are the most commonly documented [2, 3]. Additionally, up to one-third of COVID-19 inpatients present with a history of cardiovascular diseases [1]. Coexisting cardiac diseases are usually related to increased rates of infection, thromboembolism, and mortality [1, 4]. Even after the acute infection phase, individuals with a history of heart failure are at 2–4 times greater risk of heart failure exacerbation and death [4–7]. Due to their considerable power in exacerbating the severity of COVID-19-related diseases, both the acute and long-term impact of COVID-19 on cardiovascular system remain a major concern.

During the acute phase of COVID-19, both direct and indirect mechanisms have been proposed to contribute to cardiovascular injury. SARS-CoV-2 infection is initiated by binding of the receptor binding domain (RBD) of Spike surface protein to the angiotensin-converting enzyme 2 (ACE2) receptor, which is a crucial component of the renin–angiotensin–aldosterone system (RAAS) involved in regulating blood pressure and electrolyte balance [8–10]. ACE2 is highly expressed in alveolar epithelial cells and small intestinal epithelial cells, which aligns with the potential transmission pathway of SARS-CoV-2 [11]. ACE2 is also widely present in vascular endothelial cells and smooth muscle cells of various organs, including the heart, creating conditions for SARS-CoV-2 to directly infect cardiac tissues and cause acute myocardial injury and myocarditis [11]. Analysis of autopsy cases demonstrated the presence of SARS-CoV-2 in the myocardial tissues of COVID-19 patients, and in vitro cellular infection experiments confirmed the susceptibility of cardiomyocytes to SARS-CoV-2 [12–14]. Patients with different cardiac etiologies have been found to exhibit an increased expression of ACE2 in cardiac tissues, which may enhance the risk of SARS-CoV-2 infection [15, 16]. After receptor binding, the Spike protein must be cleaved by the host transmembrane protease serine 2 (TMPRSS2) to initiate membrane fusion [17]. A variety of cell-surface molecules and cellular proteases may also be involved in SARS-CoV-2 invasion [17–20]. The basal expression levels of these viral entry-associated receptors or co-factors may be a crucial determinant of SARS-CoV-2 cellular tropism; however, the correlation between their expression levels in cardiovascular disease patients and susceptibility to COVID-19 is largely unknown.

Indirect injury is mainly mediated by systemic inflammation, hypoxic injury, RAAS dysregulation, and a mismatch between myocardial supply and demand [21, 22]. While the mechanism of sustained cardiac injury after acute illness remains poorly understood. It has been postulated that the mechanisms involved in long COVID may be the consequences of physical harm to the cardiovascular system and modified physiological conditions that occurs during the acute phase of COVID-19, encompassing dysregulation of the immune system, persistent damage following inflammation, complications following critical illness, and continuous underlying viral infection [23, 24]. In addition, individuals with SARS-CoV-2 infection usually present with a multi-organ disease syndrome. The interactions between heart and other organs, such as heart-brain axis (HBA) dysfunction, may exacerbate the prognosis of patients with COVID-19 [25]. SARS-CoV-2 interferes with HBA, thereby compromising overall HBA homeostasis and leading to multiorgan complications, including arrhythmias, acute myocardial infarction, and stress cardiomyopathy [25]. It is also worth noting that the emergence of new variants of SARS-CoV-2, particularly mutations in the Spike protein, and the development of vaccines have caused changes in virulence and transmissibility of the virus [26, 27]. For example, compared with earlier Delta variant, the Omicron BA.1 subvariant was strongly attenuated in human cardiomyocyte, whereas the BA.5 subvariant showed increased replication ability [14]. Therefore, the severity of cardiac injury caused by different SARS-CoV-2 variants may vary. Although possible mechanisms for SARS-CoV-2-induced cardiac involvement have been proposed, the underlying molecular mechanisms between COVID-19 and cardiovascular diseases are not yet fully understood. A more comprehensive understanding of the interplay and molecular crosstalk between these two conditions will assist in formulating novel intervention strategies during the COVID-19 pandemic.

Integration and analysis of data through bioinformatics tools allows for more accurate prediction of the molecular pathogenesis of diseases and accelerates precision medicine. To uncover the pathogenesis of COVID-19-related cardiovascular disorders, the common differentially expressed genes of the two diseases were obtained from public databases. Based on this, the shared profiles of genes, molecular networks, and signaling pathways of COVID-19 and cardiovascular diseases were revealed, and potential therapeutic agents were predicted.

## Methods

### Data collection and processing

The second-generation high-throughput sequencing data and microarray data for illnesses were obtained from the National Center for Biotechnology Information (NCBI) Gene Expression Omnibus (GEO) datasets (http://www.ncbi.nlm.nih.gov/geo/). The GSE156754 dataset investigates the susceptibility and response of human induced pluripotent stem cell-derived cardiomyocytes (hiPSC-CMs) to SARS-CoV-2 infection, containing an infection group and a mock group, each with 3 samples [28]. The dataset GSE84796 investigates gene expression in heart failure, containing transcriptome profiles of human left ventricular free wall samples obtained from 10 end-stage heart failure patients and 7 organ donors (characteristics of clinical information of this heart failure dataset can be found in Additional file 1: Table S1) [29]. The GSE57338 dataset, containing microarray data of left ventricle samples from 177 patients with failing hearts and 136 healthy controls, was selected as a validation set for verification of the hub DEGs [30]. Datasets were accessed from GEO using the R package “GEO query” [31]. Differentially expressed genes (DEGs) from each dataset were obtained with the R package “limma”, and *P*-value < 0.05 and log2|fold change (FC)|> 1 were set as the criteria for identifying DEGs. Namely, genes meeting the criteria, *P*-value < 0.05 and FC > 2 in comparison with control, were characterized as up-regulated DEGs, whereas genes with *P*-value < 0.05 and FC < -2 compared with the control were considered to be down-regulated DEGs. Resulting DEGs were visualized by volcano plot using the online website (https://www.xiantaozi.com/). The common DEGs between the GSE156754 and GSE84796 datasets were obtained and visualized with the R packages “VennDiagram” and “ggplot2”.

### GO and KEGG enrichment analysis

To explore the shared pathogenesis between COVID-19 and cardiovascular diseases, Gene Ontology (GO) analysis, including biological process, molecular function, and cellular component, as well as Kyoto Encyclopedia of Genes and Genomes (KEGG) pathway enrichment analysis, were performed through the web-based platform DAVID (https://david.ncifcrf.gov/home.jsp) with homo sapiens genes as a background. The *P*-values obtained from DAVID’s analyses were adjusted for multiple testing using Benjamini’s correction, and a threshold value of 0.05 was applied. Redundant GO terms were then eliminated by REVIGO (http://revigo.irb.hr/) based on semantic similarity. The threshold for allowed similarity in REVIGO was set to “Medium (0.7)”, and the semantic similarity measure “SimRel” was chosen. The results of GO and KEGG enrichment analyses were visualized using web service Hiplot Pro (https://hiplot.com.cn/), a comprehensive web service for biomedical data analysis and visualization.

### Network analysis and hub genes identification

The common DEGs were used to construct the protein‒protein interaction (PPI) network using the STRING database (https://string-db.org/), with a minimum required interaction score set to medium confidence (0.4) [32], and the results were visualized using Cytoscape (v3.8.2). Hub genes and vital networks were identified using the plugins CytoHubba and MCODE in Cytoscape. Furthermore, the GeneMANIA database (https://genemania.org/) was utilized to analyze gene network of the hub genes [33].

### Identification of immune-related DEGs (ImmuneDEGs)

Immune-related genes were obtained from the ImmPort database (https://www.immport.org). ImmuneDEGs were selected and visualized by taking the intersection of the common DEGs and the immune-related genes using the R packages “VennDiagram” and “ggplot2”.

### Identification of mitochondria-related DEGs (MitoDEGs)

Mitochondrial genes were obtained from the mitochondrial protein database MitoCarta 3.0 (https://www.broadinstitute.org/mitocarta/mitocarta30-inventory-Mammalian-mitochondrial-proteins-and-pathways) [34]. MitoDEGs, which refer to nuclear genes coding proteins with mitochondrial location in this study, were obtained and visualized by taking the intersection of the common DEGs and the mitochondrial genes using the R packages “VennDiagram” and “ggplot2”.

### Immune infiltration analysis

The web-based platform CIBERSORTx (https://cibersortx.stanford.edu/) was used to estimate the expression of 22 immune cells in each sample in the heart failure cohort. Subsequently, spearman correlation analysis was performed to assess the interrelationship among the immune cells.

### Obtainment of potential key genes for cardiovascular diseases

The Comparative Toxicogenomics Database (CTD, http://ctdbase.org/) provides data on interfaces between chemicals, genetic products, biological outcomes, and diseases and contributes to the study of potential mechanisms of pharmaceutical action and disease-related environmental exposures [35]. Using the CTD database, the associations between hub genes and the risk of the onset of cardiovascular diseases, heart diseases, and vascular diseases were analyzed.

### Identification of drug candidates

The Connectivity Map (CMap, https://clue.io/), which reveals connections among pharmaceuticals, genes, and diseases [36], was utilized to screen for drug candidates. To identify potential therapeutic agents, hub DEGs were uploaded to the CMap database. According to the score, the top 3 candidate drugs (containing some drugs with the same rating) were selected as candidate drugs for the treatment of cardiac injury associated with SARS-CoV-2 infection.

### Cell culture and drug treatment

The H9c2 cells, derived from embryonic rat myocardial tissue, were used in this study to induce hypertrophy [37–39]. H9c2 cells (GNR 5) were obtained from the Shanghai Institutes for Biological Sciences, Chinese Academy of Sciences (Shanghai, China). The cells were cultured in 24-well plates at 37 ℃ in a humidified environment with 5% CO2 using Dulbecco’s modified Eagle’s medium (DMEM) supplemented with 10% fetal bovine serum (FBS; Gibco, Cat#10099–141) and 1% penicillin/streptomycin (Yeasen, Cat#60162ES76). To induce cardiomyocyte hypertrophic injury, H9c2 cells were treated with β-adrenergic agonist, isoproterenol (ISO), as described previously [40–42]. Briefly, cells were cultured with serum-free DMEM medium for 12 h and then exposed to 10, 30, and 60 μM ISO (MCE, Cat#HY-B0468) or vehicle (dimethyl sulfoxide, DMSO) for 24 h. The collected cells were subjected to qPCR analysis.

### Models of transverse aortic constriction

The animal procedure was performed in accordance with the Guide for the Care and Use of Laboratory Animals published by the US National Institutes of Health (NIH Publication No. 85–23). All experimental procedures were approved by the Animal Use Subcommittee at Soochow University, China. C57BL/6 male mice (8‒10 weeks) were purchased from JOINN Laboratories (Suzhou, China) and were used in all experiments. A mouse model of heart failure was established by transverse aortic constriction (TAC) surgery for 8 weeks, as previously described [43]. Mice were anesthetized with 2% isoflurane and incubated under controlled respiration cycled at 125‒150 breaths per minute and a tidal volume of 0.1‒0.3 mL. With the mouse supine, the aortic arch was fully exposed via the left thoracotomy. A 26G syringe needle was placed between the brachiocephalic artery and the left common carotid artery, which was followed by the ligation with a 7–0 thin thread to constrict the aortic arch to 0.44 mm in diameter. After the ligation, the syringe needle was removed immediately. Mice in the sham group underwent the same surgery without constriction of the aortic arch. All animals were given sterile saline of 1 mL containing buprenorphine and penicillin subcutaneously for half an hour prior to TAC surgery and then at 12 h intervals as appropriate.

### Echocardiography

All animals were anaesthetized with inhaled 1–2% isoflurane and imaged on a warm platform via a 40 MHz linear array transducer attached to a preclinical ultrasound system (Vevo 2100, FUJIFILM Visual Sonics, Canada) with a nominal in-plane spatial resolution of 40 μm (axial) × 80 μm (lateral) as previously described [44]. Changes of ejection fraction (EF), fractional shortening (FS), left ventricular anterior wall dimension at end-diastole (LVAW;d), left ventricular anterior wall dimension at end-systole (LVAW;s), left ventricle end diastolic inner diameter (LVID;d), left ventricle end systolic inner diameter (LVID;s), left ventricle posterior wall thickness at end-diastole (LVPW;d), left ventricle posterior wall thickness at end-systole, (LVPW;s), left ventricular diastolic volume (LV Vol;d), and left ventricular systolic volume (LV Vol;s) were determined.

### RNA extraction and qPCR

RNA isolation and qPCR were performed to analyze the mRNA expression in cells and mice failing heart tissues. Total mRNA was extracted using TRIzol (Invitrogen, Cat#15596018), followed by reverse transcription into cDNA using TaKaRa PrimeScript RT reagent Kit with gDNA Eraser (Takara, Cat#047A) according to the manufacturer’s instructions. The PCR amplifications were quantified using TaKaRa TB Green Premix Ex Taq (Takara, Cat#420A) on the StepOnePlus Real-Time PCR System (Applied Biosystems). The relative mRNA levels were calculated using the 2^−∆∆Ct^ method with Glyceraldehyde-3-phosphate dehydrogenase (GAPDH) or β-Actin as a control for standardization. The primer sequences for qPCR were clarified in Additional file 1: Table S2.

### Histological analysis

Hearts tissues were collected and fixed in 10% formalin, followed by embedded in paraffin and sectioned at 5 µm intervals following standard protocol as described previously [45]. After processing, haematoxylin and eosin (H&E) staining were performed according to standard procedures. Cardiomyocyte cross-sectional areas and collagen deposition of heart sections were determined as we previously described [44, 46].

### Malondialdehyde (MDA) assay

The relative MDA concentration in heart tissue homogenate was assessed using a Lipid Peroxidation (MDA) Assay Kit (Solarbio, Cat#BC0025) according to the manufacturer’s instructions.

### Caspase-3 activity

Caspase-3 activity in cardiac tissues was measured using a Caspase-3 Fluorescence Assay Kit (Biomol Research Laboratories, Inc., Plymouth, PA, USA) following the manufacturer’s instructions.

### Statistical analysis

Statistical analysis was performed using the bioinformatics tools mentioned above, R Studio software V4.2.1 and GraphPad Prism 6.0. The Student's *t*-test was utilized to assess the statistical significance between the two groups when the data conformed to a normal distribution. Results were considered statistically significant at *P < 0.05, **P < 0.01, ***P < 0.001, and ****P < 0.0001.

## Results

### Identification of common DEGs between COVID-19 and cardiovascular diseases

**Fig. 1:**
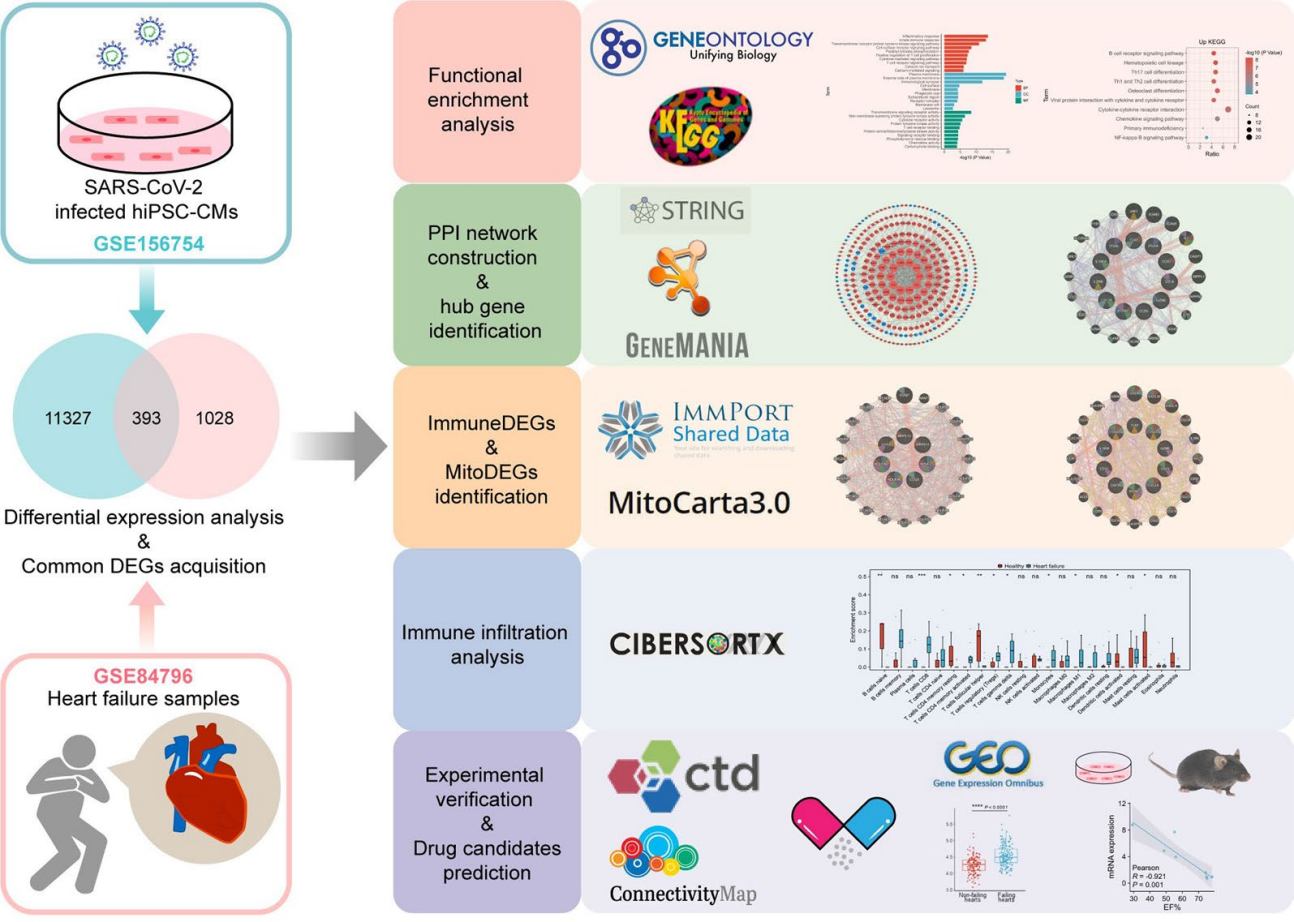
Study flowchart. Two categories of samples (SARS-CoV-2 infected hiPSC-CMs and heart failure samples) were collected from the GSE156754 dataset and GSE84796 dataset, respectively. The common differentially expressed genes (DEGs) of both datasets were identified. GO enrichment analysis, KEGG pathway analysis, protein–protein interaction (PPI) network construction, hub genes identification, immune infiltration analysis, drug candidate prediction, and experimental validation were performed on the common DEGs. hiPSC-CMs, human induced pluripotent stem cell-derived cardiomyocytes

**Fig. 2:**
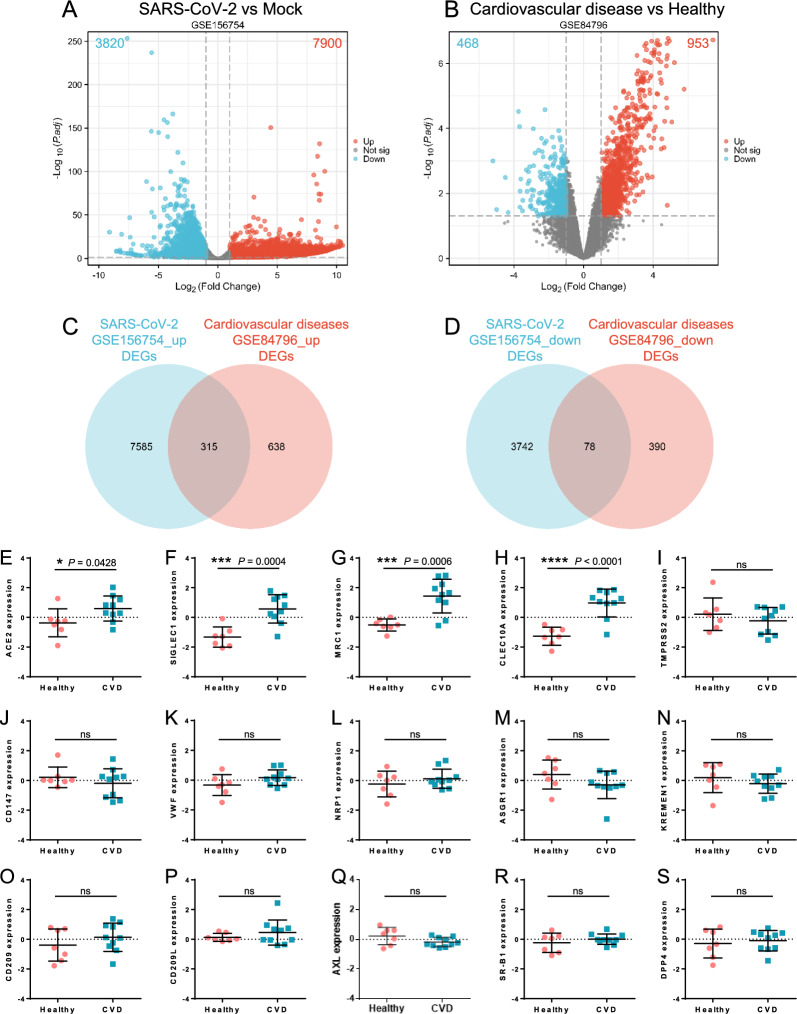
The common differentially expressed genes between SARS-CoV-2 infection and cardiovascular diseases. Volcano plot of DEGs in the SARS-CoV-2-related GSE156754 dataset (**A**) and cardiovascular diseases-related GSE84796 dataset (**B**). Red dots represent up-regulated DEGs, blue dots represent down-regulated DEGs, and gray dots represent genes that were not significantly different between the two groups. Common up-regulated (**C**) and down-regulated (**D**) DEGs in the GSE156754 dataset (blue) and GSE84796 dataset (red) were represented by venn diagrams. **E**–**S** Expression of potential receptors or co-factors for SARS-CoV-2 entry in the cardiovascular diseases-related GSE84796 dataset. See also Supplementary methods in Additional file 1 for more information on these factors. CVD, cardiovascular disease. *P < 0.05; ***P < 0.001; ****P < 0.0001; ns, no significance

The workflow diagram of this study is shown in Fig. 1. To study the interaction and crosstalk between COVID-19 and cardiovascular diseases, we downloaded and analyzed the GSE156754 and GSE84796 datasets from the GEO database. Genes meeting the criteria, *P*-value < 0.05 and fold change (FC) > 2 in comparison with control, were characterized as up-regulated DEGs, whereas genes with *P*-value < 0.05 and FC < -2 compared with the control were considered to be down-regulated DEGs. The GSE156754 dataset was applied for the identification of DEGs in SARS-CoV-2 infected cardiomyocytes. A total of 11,720 DEGs were identified, of which 7900 genes were up-regulated and 3820 genes were down-regulated (Fig. 2A). Using the GSE84796 heart failure dataset, 1421 DEGs were obtained, of which 953 genes were up-regulated and 468 genes were down-regulated (Fig. 2B). Among these DEGs, 315 common up-regulated genes and 78 common down-regulated genes were screened by the intersection of DEGs from the SARS-CoV-2 infection and heart failure datasets (Fig. 2C, D).

### Certain receptors or co-factors for SARS-CoV-2 invasion were up-regulated in individuals with cardiovascular diseases

The invasion of cells by SARS-CoV-2 is contingent upon the binding of Spike protein to receptors on the cell membrane and cleavage of Spike by cellular proteases, and the expression level of such receptors or proteases can signify the entry and dissemination of viruses as well as the clinical manifestations. To explore the distribution of receptors and entry-related co-factors for SARS-CoV-2, as well as host proteins predicted to interact with the Spike protein based on structural analysis [17–20] in individuals with cardiovascular diseases, we investigated their expression levels in myocardial tissues of heart failure patients and healthy controls (Fig. 2E–S and Additional file 1: Fig. S1. Detailed information for these factors can be found in Additional file 1: Methods). In myocardial tissues afflicted with heart failure, the expression of ACE2, the most crucial receptor for SARS-CoV-2, was significantly increased. Moreover, three other factors that have been reported to be involved in the entry of SARS-CoV-2, namely sialic acid binding Ig like lectin 1 (SIGLEC1), C-type lectin domain containing 10A (CLEC10A), and mannose receptor C-type 1 (MRC1), were also elevated. SIGLEC1 is reported to mediate the attachment of SARS-CoV-2 to antigen-presenting cells [47]. CLEC10A interacts with the Spike protein of SARS-CoV-2, and this interaction leads to a marked pro-inflammatory response in myeloid cells, which directly correlates with the severity of COVID-19 [48]. MRC1 is highly expressed in dendritic cells, monocytes and macrophages, and has a strong affinity for the SARS-CoV-2 Spike protein [49]. In addition, the expression of several genes showed a tendency of upregulation, although there was no significant difference. For instance, von willebrand factor (VWF), a marker of vascular endothelial cell phenotype, is recently found to regulate ACE2 expression in vascular endothelial cells, and may be involved in susceptibility to cardiac infections associated with the degree of oxidative stress [50]. These results suggest that patients with pre-existing cardiovascular diseases over-expressed certain receptors or co-factors for SARS-CoV-2, which may heighten the susceptibility of cardiac tissue to the virus and lead to a worse prognosis.

### GO and KEGG enrichment analysis

**Fig. 3:**
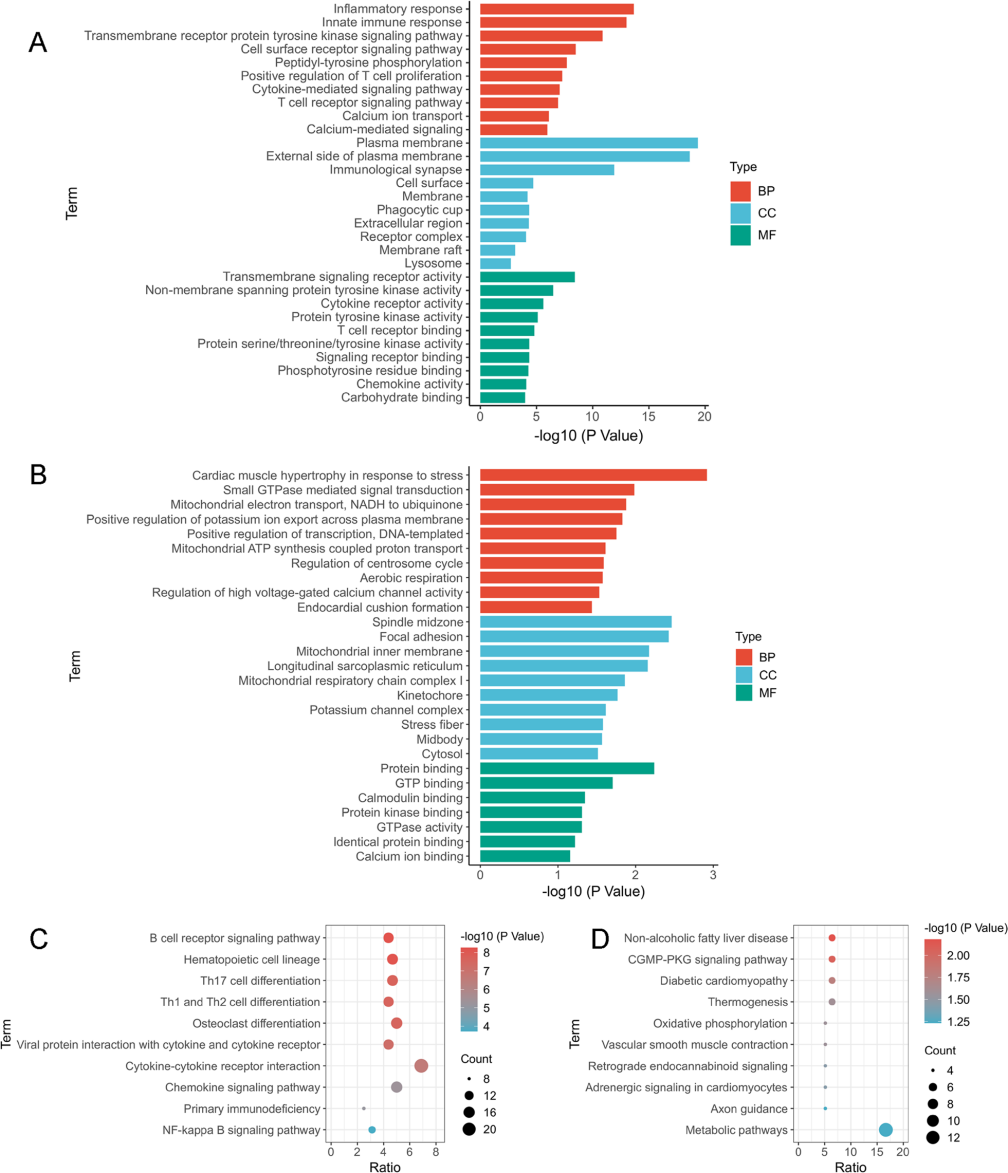
Gene Ontology and pathway enrichment analysis. GO (**A**) and KEGG enrichment analysis (**C**) of the 315 common up-regulated genes identified in SARS-CoV-2 infection and cardiovascular diseases. GO (**B**) and KEGG enrichment analysis (**D**) of the 78 common down-regulated genes identified in SARS-CoV-2 infection and cardiovascular diseases. BP, biological process; CC, cellular component; MF, molecular function

To reveal the underlying molecular mechanisms of SARS-CoV-2 infection-related cardiac injury, we performed GO enrichment and KEGG pathway analyses on the common DEGs. In biological process (BP), these common up-regulated genes were primarily responsible for inflammatory response, innate immune response, transmembrane receptor protein tyrosine kinase signaling pathway, cell surface receptor signaling pathway, peptidyl-tyrosine phosphorylation, positive regulation of T cell proliferation, cytokine-mediated signaling pathway, T cell receptor signaling pathway, calcium ion transport, and calcium-mediated signaling. In cellular component (CC), these common up-regulated genes mostly distributed in lipid bilayer membranes, such as plasma membrane, immunological synapse, phagocytic cup, receptor complex, membrane raft, and lysosome. In molecular function (MF), these common up-regulated genes were mainly involved in transmembrane signaling receptor activity, non-membrane spanning protein tyrosine kinase activity, cytokine receptor activity, protein tyrosine kinase activity, T cell receptor binding, protein serine/threonine/tyrosine kinase activity, signaling receptor binding, phosphotyrosine residue binding, chemokine activity, and carbohydrate binding (Fig. 3A). KEGG analysis showed that these common up-regulated genes were involved in B cell receptor signaling pathway, hematopoietic cell lineage, Th17 cell differentiation, Th1 and Th2 cell differentiation, osteoclast differentiation, viral protein interaction with cytokine and cytokine receptor, cytokine-cytokine receptor interaction, chemokine signaling pathway, primary immunodeficiency, and NF-κB signaling pathway (Fig. 3C). These results suggest that inflammatory response and immune activation play a crucial role in the progression of COVID-19 and cardiovascular diseases.

For these common down-regulated genes, among the biological process, DEGs were mainly enriched in cardiac muscle hypertrophy in response to stress, small GTPase mediated signal transduction, mitochondrial electron transport, NADH to ubiquinone, positive regulation of potassium ion export across plasma membrane, positive regulation of transcription, DNA-templated, mitochondrial ATP synthesis coupled proton transport, regulation of centrosome cycle, aerobic respiration, regulation of high voltage-gated calcium channel activity, and endocardial cushion formation. In cellular component, these common down-regulated genes were mostly localized in spindle midzone, focal adhesion, mitochondria, longitudinal sarcoplasmic reticulum, and so forth. In molecular function, these common down-regulated genes were primarily concentrated in protein binding, GTP binding, calmodulin binding, protein kinase binding, GTPase activity, identical protein binding, and calcium ion binding (Fig. 3B). In terms of the KEGG pathway, the common down-regulated genes were enriched in pathways of non-alcoholic fatty liver disease, cGMP-PKG signaling pathway, diabetic cardiomyopathy, thermogenesis, oxidative phosphorylation, vascular smooth muscle contraction, retrograde endocannabinoid signaling, adrenergic signaling in cardiomyocytes, axon guidance, and metabolic pathways (Fig. 3D). These results showed that mitochondrial dysfunction, metabolic dysregulation, and cytokinesis failure play a crucial role in the progression of COVID-19 and cardiovascular diseases. In addition, these functional enrichment analyses also indicated that SARS-CoV-2 infection had an impact on pathways associated with the heart, such as diabetic cardiomyopathy and adrenergic signaling in cardiomyocytes.

### Protein‒protein interaction network analysis and hub genes identification

**Fig. 4:**
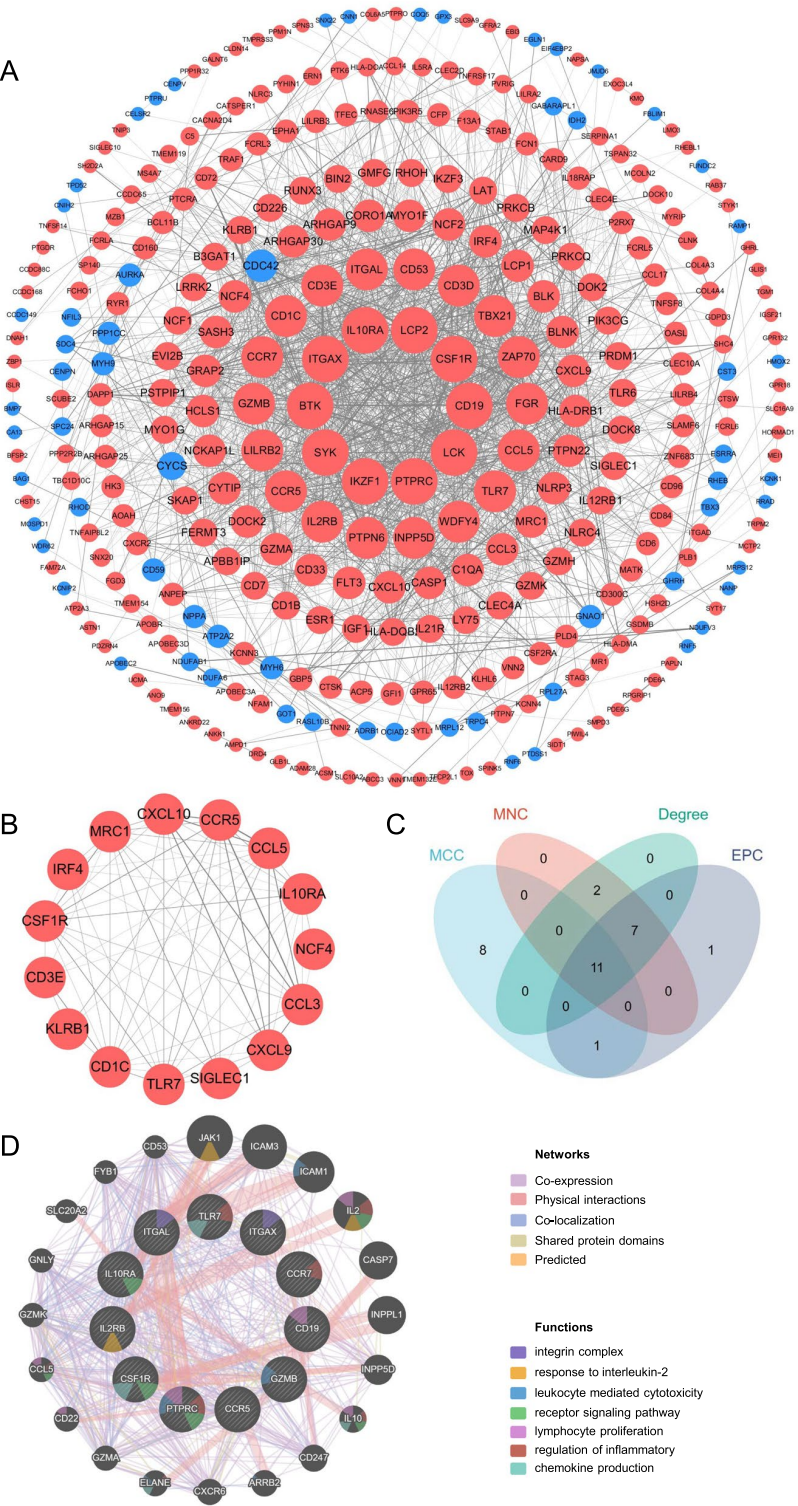
Protein‒protein interaction (PPI) network analysis and hub genes identification. **A** PPI network for common DEGs that are shared by SARS-CoV-2 infection and cardiovascular diseases. Red nodes represent up-regulated DEGs, and blue nodes represent down-regulated DEGs. The size of the node responds to its degree in the network. The thickness of the edges is related to the strength of the interaction. **B** A key cluster with 15 genes was further chosen by MCODE plugin in Cytoscape. **C** The 11 hub genes identified according to four algorithms (MCC, MNC, degree, and EPC) of CytoHubba plugin in Cytoscape (see also Table 1). **D** The gene network and functional analysis of hub genes were generated using GeneMANIA. The inner circle stands for hub genes, and the outer circle refers to the corresponding reciprocal genes. Colors of nodes represent gene function annotations, which refer to GO terms enriched among the genes in the network displayed by GeneMANIA. Colors of edges represent interaction based on co-expression, physical interactions, co-localization, shared protein domain, or predicted interaction

The 393 common DEGs were uploaded into STRING to construct the PPI network, and the generated file was imported into Cytoscape for visualization (Fig. 4A). The MCODE plugin was used to recognize key clusters, and a significant module consisting of 15 nodes and 72 edges was identified, as depicted in Fig. 4B. The genes involved in the module were IL10RA, TLR7, CD1C, CCL5, CCL3, CXCL10, CCR5, IRF4, CSF1R, MRC1, CXCL9, KLRB1, CD3E, SIGLEC1, and NCF4. Then, using the CytoHubba plugin based on four algorithms, 11 genes (PTPRC, CD19, ITGAX, GZMB, IL10RA, TLR7, CSF1R, CCR7, CCR5, ITGAL, and IL2RB) were identified as hub genes and considered the core targets of COVID-19 and cardiovascular diseases (Fig. 4C and Table 1). A gene network was further constructed using GeneMANIA. Gene functional annotation revealed that these hub genes are all associated with innate immune and inflammatory response, primarily involved in leukocyte mediated cytotoxicity, response to interleukin-2, receptor signaling pathway, lymphocyte proliferation, regulation of inflammation, and chemokine production (Fig. 4D).

**Table 1:** The top 20 hub genes analyzed by CytoHubba

Rank	MCC	MNC	Degree	EPC
1	PTPRC	PTPRC	PTPRC	PTPRC
2	CD19	LCP2	LCP2	LCP2
3	ITGAX	SYK	SYK	SYK
4	GZMB	ITGAX	ITGAX	CD19
5	IL10RA	CD19	CD19	IKZF1
6	CCL5	LCK	LCK	ITGAX
7	CXCL10	BTK	BTK	LCK
8	CD1C	IKZF1	IKZF1	BTK
9	CCL3	CSF1R	CSF1R	IL10RA
10	TLR7	IL10RA	IL10RA	GZMB
11	MRC1	GZMB	GZMB	CCR7
12	CSF1R	CCR7	CCR7	ITGAL
13	CCR7	ZAP70	ZAP70	ZAP70
14	CXCL9	IL2RB	IL2RB	IL2RB
15	CCR5	ITGAL	ITGAL	TLR7
16	IRF4	CCR5	CCR5	CSF1R
17	TBX21	TLR7	TLR7	CCR5
18	GZMA	FGR	FGR	CD3E
19	ITGAL	CD3E	CD3E	CCL5
20	IL2RB	LILRB2	LILRB2	CD3D

MCC, MNC, degree, and EPC represent different algorithms of CytoHubba plugin in Cytoscape

**Fig. 5:**
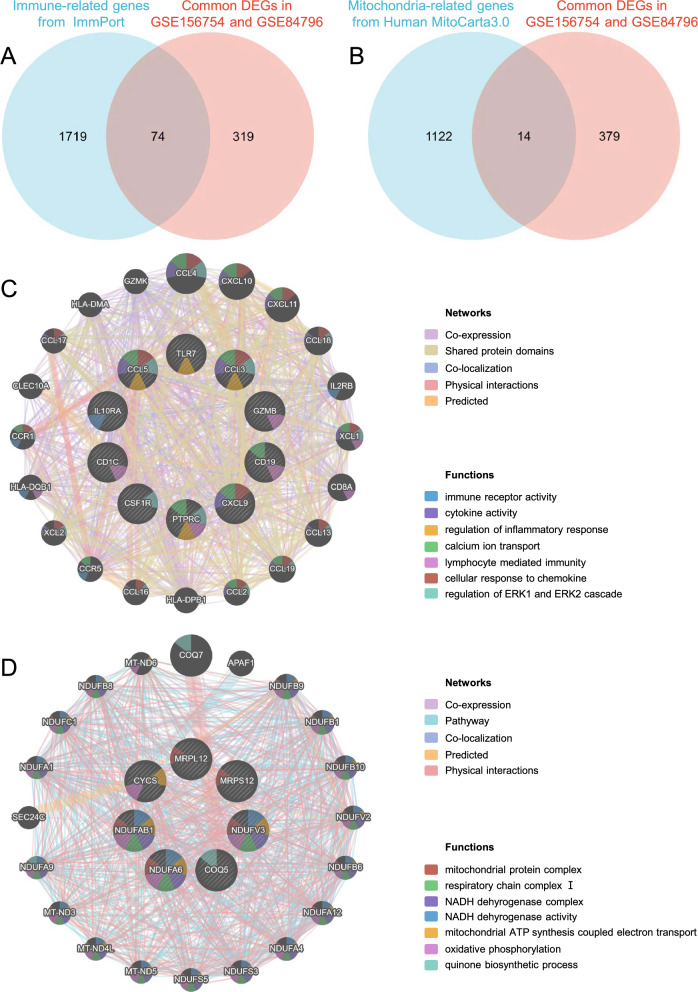
Network and functional analysis of ImmuneDEGs and MitoDEGs. Venn diagram showing 74 ImmuneDEGs (**A**) and 14 MitoDEGs (**B**). ImmuneDEGs and MitoDEGs were submitted to the STRING database and 10 hub ImmuneDEGs and 7 hub MitoDEGs were identified using the CytoHubba plugin in Cytoscape. Then, the 10 hub ImmuneDEGs (**C**) and 7 hub MitoDEGs (**D**) were uploaded in the GeneMANIA database for the further gene network analysis. The inner circle stands for hub genes, and the outer circle refers to the corresponding reciprocal genes. Colors of nodes represent gene function annotations, which refer to GO terms enriched among the genes in the network displayed by GeneMANIA. Colors of edges represent interaction based on co-expression, shared protein domain, co-localization, physical interactions, pathway, or predicted interaction. ImmuneDEGs, immune-related DEGs; MitoDEGs, mitochondria-related DEGs

In addition, as the functional enrichment results above suggested, immune activation and mitochondrial dysfunction were closely associated with the course of both diseases. Therefore, we conducted further scrutiny of the correlation between genes related to immunity or mitochondria. Immune-related genes were searched from the ImmPort database, and genes overlapping with common DEGs were selected as ImmuneDEGs. Totally, 74 ImmuneDEGs (67 up-regulated and 7 down-regulated) were obtained (Fig. 5A). Then, 10 hub ImmuneDEGs were identified using CytoHubba, including PTPRC, IL10RA, TLR7, CCL5, CCL3, CXCL9, CD19, CD1C, CSF1R, and GZMB, most of which were also identified as the aforementioned hub genes. These hub ImmuneDEGs were further imported into GeneMANIA to construct a gene network (Fig. 5C). IL10RA has immune receptor activity [51]. CCL5, CCL3, and CXCL9 are chemokines with cytokine activity and are involved in the cellular response to chemokine. In addition, these hub ImmuneDEGs are mainly enriched in biological processes such as regulation of ERK1 and ERK2 cascade, lymphocyte mediated immunity, regulation of inflammatory response, and calcium ion transport.

Similarly, mitochondria-associated genes, mainly referring to nuclear genes coding proteins with mitochondrial location, were accessed from the MitoCarta3.0 database, and genes that were overlapping with shared DEGs were chosen as MitoDEGs. After intersection with the common DEGs, 14 genes (3 up-regulated and 11 down-regulated) were selected as MitoDEGs (Fig. 5B). These genes were then subjected to CytoHubba analysis, which highlighted 7 hub MitoDEGs, namely NDUFAB1, NDUFA6, NDUFV3, MRPS12, MRPL12, CYCS, and COQ5. Hub MitoDEGs were then used to construct a gene network via GeneMANIA (Fig. 5D). These hub MitoDEGs are mainly localized to mitochondrial protein complex, such as NDUFAB1, NDUFA6 and NDUFV3 are members of the NADH dehydrogenase complex with NADH dehydrogenase activity [52, 53]. In terms of biological processes, NDUFAB1, NDUFA6, NDUFV3, and CYCS are involved in mitochondrial ATP synthesis coupled electron transport, and COQ5 is involved in quinone biosynthesis process [54].

### Immune cell infiltration in cardiovascular diseases

**Fig. 6:**
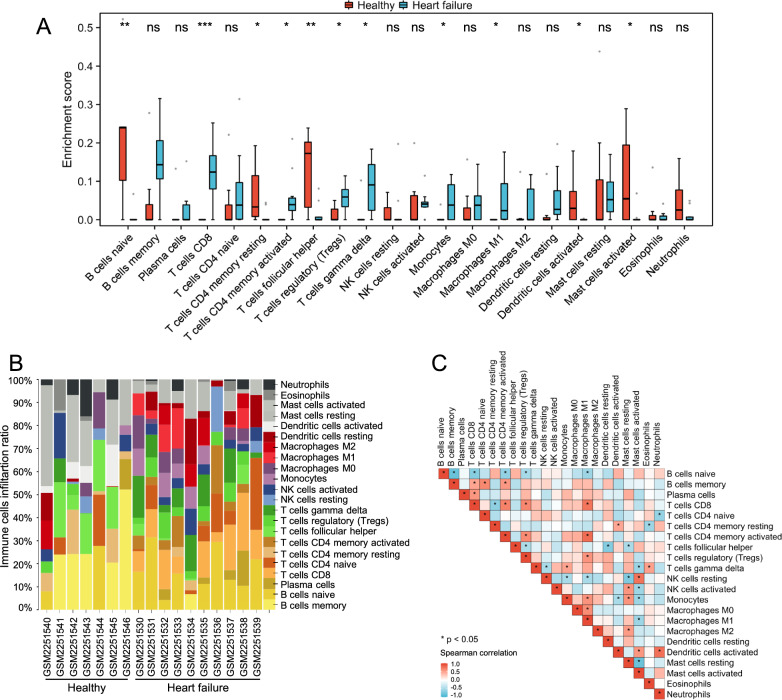
Immune infiltration analysis. **A** Immune cell infiltration analysis in the cardiovascular diseases-related GSE84796 dataset. **B** Stacked bar chart showing the percentage of immune cells in each sample. **C** Heat map showing the correlation between immune cells. *P < 0.05; **P < 0.01; ***P < 0.001; ns, no significance

Considering the vital role of immune activation in COVID-19-related cardiovascular diseases, we conducted a detailed analysis of the immune microenvironment in the cardiac tissue of patients with cardiovascular diseases. Using the CIBERSORTx algorithm, we examined the infiltration of 22 different kinds of immune cells and compared them between the cardiovascular diseases group and the control group. Our findings showed that there were significant differences in the myocardial infiltration of 11 immune cell types between the two groups. Specifically, CD8 ^+^ T cells, activated memory CD4 ^+^ T cells, Tregs, γδT cells, monocytes, and M1 macrophages were much more abundant in the cardiovascular diseases group, whereas naive B cells, resting memory CD4 ^+^ T cells, follicular helper T cells, activated dendritic cells, and activated mast cells were more abundant in the control group (Fig. 6A, B). Further analysis also revealed various correlations among infiltrating immune cells (Fig. 6C).

### Prediction of candidate drugs for COVID-19-related cardiovascular diseases

To anticipate efficacious agents for the therapy of COVID-19-related cardiovascular diseases, the 11 hub DEGs were submitted to the cMAP database. The results of drug candidates were generated based on the absolute enrichment score (Table 2). The analysis showed that actarit, raltegravir, epigallocatechin, doxylamine, lidocaine, tetrabenazine, escitalopram, desoxypeganine, and valproic-acid were the top 9 candidate drugs. These drugs represent the potential drugs for COVID-19-related cardiovascular diseases.

**Table 2:** Candidate drugs predicted with the common hub genes

Rank	Drug name	Score	Description
1	Actarit	− 99.93	Interleukin receptor agonist
2	Raltegravir	− 99.93	HIV integrase inhibitor
3	Epigallocatechin	− 99.93	Nitric oxide synthase inhibitor
4	Doxylamine	− 99.93	Histamine receptor antagonist
5	Lidocaine	− 99.89	Histamine receptor agonist
6	Tetrabenazine	− 99.89	Vesicular monoamine transporter inhibitor
7	Escitalopram	− 99.89	Selective serotonin reuptake inhibitor (SSRI)
8	Desoxypeganine	− 99.89	Acetylcholinesterase inhibitor
9	Valproic-acid	− 99.86	HDAC inhibitor

### Experimental validation of hub genes

**Fig. 7:**
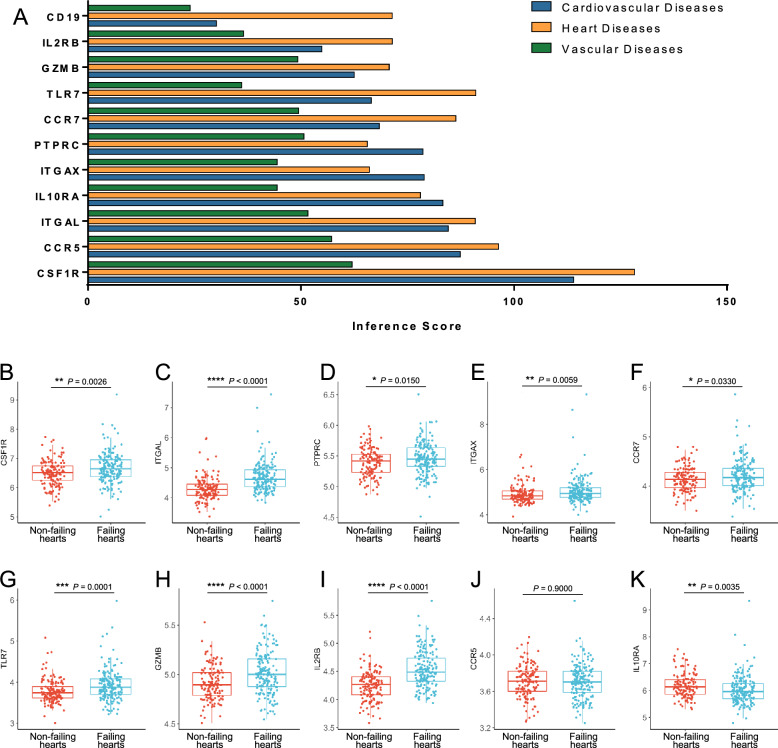
Relationship between hub genes and cardiovascular diseases and validation of hub genes in heart failure patients. **A** Analysis of the relationship between hub genes and cardiovascular diseases, heart diseases, and vascular diseases based on the CTD database. **B**–**K** Expression of hub genes in the validation set GSE57338. *P < 0.05; **P < 0.01; ***P < 0.001; ****P < 0.0001

To strengthen the clinical relevance of hub DEGs, we conducted an analysis on the association between hub genes and clinical features. The CTD database was utilized to forecast the links between hub DEGs and cardiovascular, heart, and vascular diseases. The results demonstrated that CSF1R, CCR5, ITGAL, IL10RA, and ITGAX had the highest association with cardiovascular diseases, and CSF1R, CCR5, TLR7, ITGAL, and CCR7 showed the highest correlation with heart diseases, while CSF1R, CCR5, ITGAL, PTPRC, and CCR7 showed the highest correlation with vascular diseases (Fig. 7A). To verify the expression of the top 10 cardiovascular diseases-related hub genes shown in Fig. 7A, we analyzed the GSE57338 dataset to assess their expression in heart failure patients. The results revealed that most of the genes, including PTPRC, ITGAX, GZMB, TLR7, CSF1R, CCR7, ITGAL, and IL2RB, exhibited significantly elevated expression levels in failing hearts as compared to non-failing hearts (Fig. 7B–K).

**Fig. 8:**
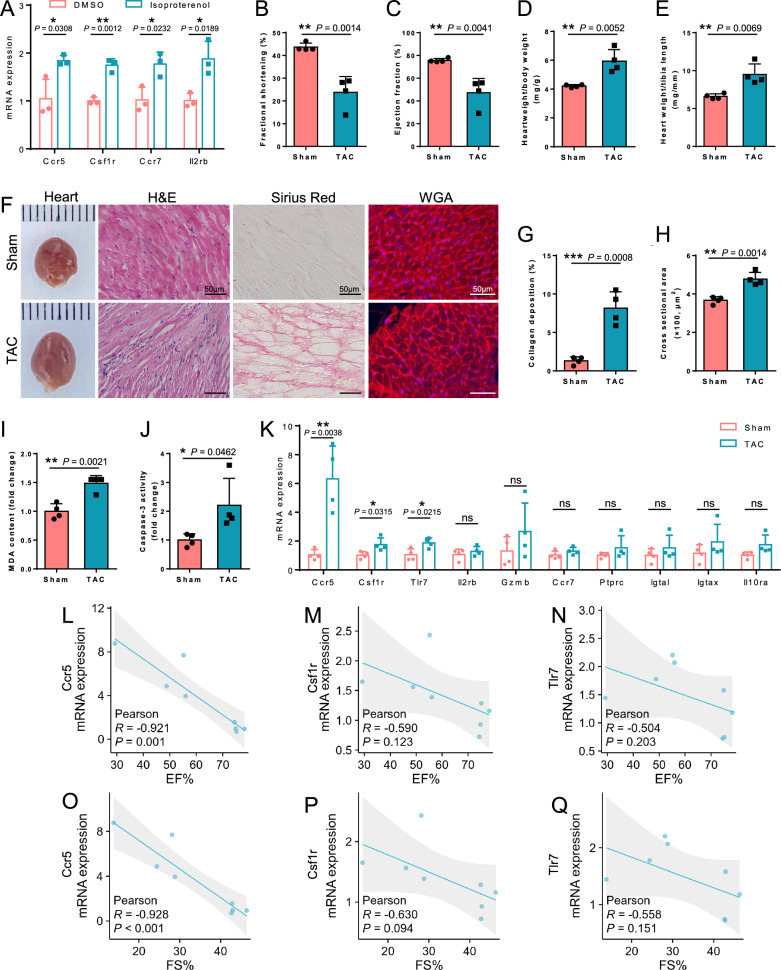
Experimental verification of the expression of hub genes in cardiac injury. **A** H9c2 cells treated with DMSO or 60 μM isoproterenol for 24 h were performed to analyze mRNA expression levels of hub genes. Values represent means ± SD (n = 3 biologically independent samples). **B**–**Q** Characterization of physiological and cardiac features of transverse aortic constriction (TAC) induced heart failure and confirmation of hub genes expression. Heart failure was induced by TAC at a 0.44 mm diameter for 8 weeks. Cardiac function (**B** and **C**) was determined in the heart. Hearts were collected and processed for anatomical and histologic analysis. Heart weight/body weight (**D**) and heart weight/tibia length (**E**). **F** Representative image of whole heart, and histological picture of H&E staining, Sirius red staining, and fluorescence-conjugated wheat germ agglutinin (WGA) staining for heart. Bars, 50 μm. Quantification for collagen deposition (**G**) and cardiomyocyte cross-sectional areas (**H**). **I** The levels of malondialdehyde (MDA) in heart lysates. **J** Apoptosis was determined based on caspase-3 activity. **K** The expression levels of mRNA of hub genes were confirmed in sham and TAC mice. Values represent means ± SD (n = 4 mice). **L**–**Q** Correlations between Ccr5, Csf1r, and Tlr7 mRNA expression levels and cardiac functional parameters. *R* denotes the correlation coefficient between the variables and *P* denotes the statistical *P*-value between the variables, which were obtained by Pearson correlation analysis. All qPCR graphs show gene expression normalized to Glyceraldehyde-3-phosphate dehydrogenase (Gapdh). *P < 0.05; **P < 0.01; ns, no significance

Subsequently, isoproterenol (ISO)-stimulated cardiomyocyte hypertrophic injury in H9c2 cells and a transverse aortic constriction-induced heart failure model in mice were used to further verify the expression of hub genes [37–39]. First, different concentrations of ISO were used to establish an in vitro cell model using H9c2 cells. The results showed that, treatment of H9c2 cells with 60 μM ISO for 24 h induced a significant up-regulation of the expression of cardiac hypertrophy markers, atrial natriuretic peptide (ANP) and myosin heavy chain β (β-MHC), and had no significant effect on cell viability (Additional file 1: Fig. S2). Additionally, in ISO-treated H9c2 cells, the mRNA levels of Ccr5, Csf1r, Ccr7, and Il2rb were also remarkably raised (Fig. 8A). The remaining genes were difficult to detect, probably due to their low expression in H9c2 cells. Similarly, in ISO-stimulated hiPSC-CMs, which exhibits an arrhythmic phenotype [55, 56], mRNA expression levels of CCR5, CSF1R, TLR7, IL2RB, and IGTAX were upregulated (Additional file 1: Fig. S3).

In 8-week TAC mice, echocardiographic measurement demonstrated that ejection fraction and fractional shortening were decreased in TAC mice (Fig. 8B, C and Additional file 1: Table S3). In line with this, histological and anatomical analyses revealed that TAC mice exhibited noticeable changes associated with myocardial hypertrophy and fibrosis. These changes included increased inflammatory cell infiltration, collagen deposition, enlarged cross-sectional areas of cardiomyocytes, and enlargement of the heart size (Fig. 8D–H). Accordingly, analysis of gene expression demonstrated upregulation of mRNA levels related to myocardial hypertrophy (ANP and β-MHC) and fibrosis (Col3a1) in the hearts of TAC mice compared to the sham group (Additional file 1: Fig. S4). In addition, myocardial oxidative stress and apoptosis were further evaluated. The results showed increased production of MDA, the lipid peroxidation marker, and elevated caspase-3 activity, in the heart tissues of TAC mice (Fig. 8I, J). Taken together, these results demonstrate that TAC surgery induces hypertrophy and heart failure in mice. As shown in Fig. 8K, mRNA levels of three candidate genes (Ccr5, Csf1r, and Tlr7) were significantly increased in heart failure mice. Meanwhile, the remaining hub genes, while not displaying statistical significance, exhibited a discernible trend of increased expression in heart failure mice. Then, the relationship between the above three hub genes (Ccr5, Csf1r, and Tlr7) and cardiac function was further investigated. The expression of Ccr5 was significantly negatively correlated with EF% (R = − 0.921; *P* = 0.001) and FS% (R = − 0.928; *P* < 0.001); while the expression of Csf1r and Tlr7 was slightly negatively correlated with EF% and FS% (Fig. 8L–Q). These findings suggest that the up-regulated expression of Ccr5, Csf1r, and Tlr7 in the myocardial tissues is related to the decline of cardiac function in heart failure mice.

## Discussion

During the COVID-19 pandemic, it has been recognized that SARS-CoV-2 infection can induce serious or even fatal cardiac complications [1, 57]. Moreover, patients with pre-existing cardiovascular diseases have poorer outcomes after developing COVID-19 [1, 4]. However, there is limited research on the shared molecular mechanisms between COVID-19 and cardiovascular diseases. In the present study, we identified 315 common up-regulated DEGs and 78 common down-regulated DEGs between COVID-19 and cardiovascular diseases. Functional enrichment analysis showed that mitochondrial dysfunction, metabolic abnormality, immune activation, and inflammatory response were the most important features of COVID-19 and cardiovascular diseases. In addition, enrichment of cardiovascular-related pathways, such as diabetic cardiomyopathy, adrenergic signaling in cardiomyocytes, and vascular smooth muscle contraction, may reflect the occurrence of cardiovascular injury in COVID-19 patients. Representative aspects of the interplays between COVID-19 and cardiovascular diseases highlighted in this study are further discussed below.

The mitochondrion plays a vital role in the maintenance of intracellular homeostasis. Mitochondria are responsible for a series of essential cellular processes, for instance, ATP synthesis, anabolism and catabolism, fatty acid oxidation, calcium ion storage, modulation of innate immune signaling and inflammatory response, and regulation of cell death [58]. Our results reveal that genes associated with energy metabolism in mitochondria are down-regulated predominantly in both COVID-19 and cardiovascular diseases. These findings are consistent with results from multiple actual infection systems or patients. For instance, patients with COVID-19 exhibit notable mitochondrial dysfunction as well as metabolic disturbances marked by an upsurge in glycolysis in peripheral blood mononuclear cells (PBMCs) [59]. SARS-CoV-2 can also cause transcriptional downregulation of the mitochondrial respiratory chain complex and ATP synthesis in hiPSC-CMs [60]. Our and these previous studies demonstrate that mitochondrial dysfunction and metabolic alteration are involved in COVID-19. Mitochondrial metabolic abnormalities have also been shown to play a critical role in various cardiovascular disorders [61]. Under physiological conditions, cardiomyocytes critically depend on fatty acid-driven oxidative phosphorylation for ATP synthesis [62]. In a variety of cardiomyopathies, cardiomyocytes are accompanied by ATP exhaustion and increase of reactive oxygen species (ROS), which may directly compromise myocardial function [63]. In general, the increase in ROS and the consumption of ATP appears to be associated with impaired homeostasis of certain metabolites and co-factors that are beneficial for proper mitochondrial function, as well as impairment of mitochondrial dynamics and abnormal restructuring of mitochondrial and mitochondrial membrane structures [64]. Thus, mitochondria and mitochondrial proteins are a promising set of pharmacological targets for the search of new molecular targets and drugs to combat cardiovascular diseases [64, 65].

In addition to regulating energy metabolism, maintaining intracellular Ca^2+^ homeostasis is also a core function of mitochondria. According to the outcomes of our GO analysis, disturbances in Ca^2+^ homeostasis are present in both diseases, as evidenced by the enrichment of DEGs in calcium ion transport, calcium-mediated signaling, and calcium channel activity. Calcium ion acts as an intracellular messenger and regulator of numerous cellular processes and is central to overall cardiac activity [66]. For example, Ca^2+^ flux is crucial for cardiomyocytes and vascular smooth muscle cells because both cardiac and arterial contractions rely on instantaneous changes in cellular Ca^2+^ concentrations [66]. SARS-CoV-2-encoded E and ORF3a proteins function as viroporins, which form Ca^2+^ channels at the mitochondria-ER contact sites within infected cells [67, 68]. The resultant alteration in intracellular Ca^2+^ homeostasis subsequently triggers the inflammatory cascade, leading to a cytokine storm and tissue injury. Extensive and unreversible mitochondrial dysfunction, culminating in altered permeability of the mitochondrial membrane, has a pivotal role in mitochondrial-regulated cell death, including apoptosis, regulatory necrosis, and pyroptosis [69]. The irreversible death of damaged cells is an essential trigger for the development of cardiovascular diseases, including atherosclerosis, myocardial infarction, and heart failure.

In addition, mitochondria serve as an important innate immune antiviral platform by housing the mitochondrial antiviral signaling protein (MAVS) to act as a central hub for antiviral signaling [70]. Viral components may be involved in the regulation of mitochondrial function through interactions with proteins with mitochondrial localization. For example, a recent study based on a computational approach predicting the interaction of Spike protein and ACE2-related proteins found that Spike protein may interact with neurolysin (NLN) and thimet oligopeptidase (THOP1), which have high structural similarity to ACE2 and have mitochondrial localization [18]. NLN and THOP1 are closely-related zinc metallopeptidases, NLN plays a crucial role in the assembly of respiratory chain supercomplexes [71], and both NLN and THOP1 play key physiological functions in the regulation of energy metabolism [72]. Interestingly, the mRNA expression level of THOP1 were downregulated in cardiac tissues from heart failure patients, which could be related to impaired cardiac energy metabolism in heart failure individuals (Additional file 1: Fig. S1B). The potential interaction between Spike protein and these proteins, which are localized in mitochondria and play a role in regulating energy metabolism, offers insights into how SARS-CoV-2 causes damage to cardiac tissue by disrupting mitochondrial function. The shared cardiac mitochondrial dysfunction and metabolic profile of COVID-19 and cardiac diseases imply that SARS-CoV-2 infection may potentially contribute to worsening clinical symptoms of cardiovascular diseases.

Mitochondria and immune system are inextricably interrelated, and mitochondrial function is crucial for modulating the immune response and resolution during recovery after cardiac injury [73, 74]. Innate immunity serves as the first barrier against viruses, while an imbalanced immune system can also trigger life-threatening inflammatory responses. Reduced innate antiviral defenses and “cytokine storm” are thought to be the pathological mechanisms of COVID-19 and the sequelae of the circulatory system in COVID-19, with monocytes and macrophages being the primary contributors [75, 76]. Evidence from clinical studies indicates that the severity of COVID-19 is positively associated with chemokine and inflammatory cytokine levels in the plasma of patients, such as TNF-α, IL-1β, IL-6, CCL2, CCL8, and CXCL9 [76, 77]. Additionally, long COVID patients experience persistent immunological dysfunction, including absence of naive T and B cells and high levels of inflammatory mediators in their plasma, even 8 months after infection [24, 78]. Chronic inflammatory disorders are also risk factors associated with a variety of cardiovascular diseases [79, 80]. For example, pro-inflammatory response driven by TLR signaling is a critical mechanism in the pathogenesis of atherosclerosis [81]. As atherosclerosis progresses, inflammation and immune responses promote instability of the arterial lesions, which leads to lethal consequences such as myocardial infarction. In this study, analysis of the immune microenvironment revealed an increase of monocytes and M1 macrophages and a reduction of naive B cells in myocardial tissues from individuals with cardiovascular diseases, exhibiting an immune cell fraction profile similar to that of COVID-19. Since inflammation is an important player in both COVID-19 and cardiovascular diseases, we hypothesize that abnormal activation of the immune system and excessive systemic inflammatory response, rather than a direct cell-killing effect caused by SARS-CoV-2 infection, may be triggers for cardiac involvement.

Additionally, the hub genes associated with the common pathogenesis of COVID-19 and cardiovascular diseases identified in this study, namely PTPRC, ITGAX, GZMB, IL10RA, TLR7, CSF1R, CCR7, CCR5, ITGAL, and IL2RB, are all associated with innate immune and inflammatory responses. Integrin signaling has been shown to regulate endothelial phenotype, leukocyte homing, and smooth muscle fibro proliferative remodeling, thereby contributing to the development of cardiovascular diseases [82]. The discovery of ITGAX and ITGAL, members of the integrin family, suggests that these integrin regulatory mechanisms may be the pathway by which SARS-CoV-2 drives its adverse effects on the cardiovascular system. Colony-stimulating factor 1 receptor (CSF1R) is important for proliferation, differentiation and survival of mononuclear phagocytes and has been identified as a key gene in COVID-19-related cardiovascular diseases [83, 84]. The PTPRC gene, which encodes the protein tyrosine phosphatase receptor type C, is critical for regulating antigen receptor signaling in T and B cells. PTPRC is revealed as a new biomarker for SARS-COV-2 pathogenesis through the analysis of various transcriptomic datasets from COVID-19 patients [85]. Toll-like receptor 7 (TLR7), a member of the toll-like receptor family, has an essential role in pathogen recognition and immune system activation. SARS-CoV-2 can be recognized by TLR7 and activate the MyD88-dependent NF-κB pathway, resulting in elevated production of pro‐inflammatory cytokines [86, 87]. Additionally, TLR7 activation has been linked to the acceleration of cardiovascular pathology [88]. GZMB encodes a serine protease, which is crucial for cytotoxic T lymphocyte-mediated cell death. SARS-CoV-2 infection triggers a strong cytotoxic T-cell immune response [89]. Similarly, our immune infiltration analysis showed significant activation of CD8 ^+^ T cells in the cardiac tissues of heart failure patients (Fig. 6). GZMB also plays a role in matrix remodeling and cardiac fibrosis [90]. The interleukin 10 (IL-10) receptor, IL10RA, mediates the immunosuppressive signal of the anti-inflammatory cytokine, IL-10, which limits excessive tissue disruption caused by inflammation [51]. The elevation of IL-10 receptors in COVID-19 and cardiovascular diseases appears to be a mechanism by which the body regulates pathological consequences caused by inflammatory response. IL2RB, a receptor for IL-2, is involved in T cell-mediated immune responses and plays a fundamental role in the development of coronary artery disease [91]. Chemokines and chemokine receptors play a great function in controlling the degree of immune cell infiltration. The higher expression levels of CCR5, CCR7 and their ligands are associated with the development and progression of cardiovascular diseases, such as atherosclerosis [92]. These results further strengthen the potential role of the hub genes we identified in the pathogenesis of COVID-19-related cardiovascular diseases.

Since the pandemic, the development of vaccines against SARS-CoV-2 has progressed rapidly, and approved vaccines, mainly including inactivated, mRNA, protein subunit, and adenoviral vector vaccines, have been highly effective in preventing COVID-19, particularly severe disease [26, 93]. However, as the protective effect of vaccines diminishes due to viral variants, the passage of time since vaccination, and waning immunity [26, 27], there remains an urgent need for new antiviral therapies to combat the pandemics. Currently, several antiviral drugs, such as paxlovid, immunomodulatory drugs, and anti-SARS-CoV-2 neutralizing monoclonal antibodies (i.e., bamlanivimab, etesevimab, sotrovimab, casirivimab, and imdevimab, which block infection of target cells while invoking phagocytosis and elimination of the virus), have been proposed as potential treatments for COVID-19 [94–96]. Despite the promise of these agents, their high cost renders them impractical for large-scale implementation. Moreover, the multiple complications accompanying SARS-CoV-2 infection limit the currently available options, and the safety of drug administration needs to be considered more thoroughly. Thus, a comprehensive analysis of the common pathogenesis between COVID-19 and cardiovascular diseases may yield valuable insights for future personalized treatments for specific populations. Here, we propose potential drugs that may be used to treat COVID-19-associated cardiac injury. For example, epigallocatechin, a compound in green tea, has been reported to have anti-SARS-CoV-2 and cardioprotective efficacy [97, 98]. Epigallocatechin may exert protective effect on mitochondrial function by preventing mitochondrial ROS induced by SARS-CoV-2 or cardiovascular disease via its broad antioxidant activity [97, 98]. Lidocaine, a local anesthetic agent, has been approved for the treatment of cardiac arrhythmias owing to its remarkable anti-inflammatory properties. Recent research has revealed that lidocaine effectively modulates the inflammatory response induced by the SARS-CoV-2 infection [99]. Actaris, an anti-rheumatic drug, is employed to rectify immunological disorders through immunomodulation [100]. Raltegravir acts as an inhibitor of HIV-1 integrase [101]. The aforementioned drugs exert their effects by modulating immune response, inflammation, or mitochondrial function, which are also common pathological manifestations of COVID-19 and cardiovascular diseases, and additional validations are necessary to further investigate their potential applications. These findings suggest a deeper understanding of the shared molecular pathogenic mechanisms between COVID-19 and cardiovascular diseases could be strategically useful in developing therapeutic targets in the treatment of cardiac injury associated with SARS-CoV-2 infection.

However, there are limitations to this study due to data and sample restrictions. Specifically, the investigation relied on data obtained solely from virus-infected cardiomyocytes or patients with heart failure, rather than those with both conditions. And we were unable to acquire cardiac tissue samples from patients with COVID-19 and cardiovascular diseases to confirm the expression of key genes. Given the limitations, it is necessary to collect more information on patients with cardiac injury associated with SARS-CoV-2 infection and explore the relationship between viral infection and the occurrence of cardiac involvement in the future.

## Conclusions

This study is critical for understanding SARS-CoV-2-host interactions, the mechanisms underlying SARS-CoV-2-induced cardiac injury, and the factors contributing to poorer outcomes in COVID-19 patients with pre-existing cardiovascular diseases. These key genes identified in this study, particularly immune-inflammation and mitochondrial metabolism-related genes, along with the signaling pathways they control, may guide the clinical management of COVID-19 patients and facilitate the development of new and effective therapeutic strategies.

### Supplementary Information

**Additional file 1: Figure S1.** Expression of host proteins predicted to interact with SARS-CoV-2 Spike protein based on structural analysis in the cardiovascular diseases-related GSE84796 dataset. **Figure S2.** Effect of isoproterenol (ISO) on H9c2 cells. **Figure S3.** Experimental verification of hub genes expression in isoproterenol induced hiPSC-CMs. **Figure S4.** Expression of cardiac hypertrophy and fibrosis markers in heart tissues of TAC mice. **Table S1.** Characteristics of heart tissue samples from heart failure dataset used in this study. **Table S2.** Primers used in this study. **Table S3.** Parameters of cardiac functions in two groups of mice.

## Data Availability

The datasets (GSE156754, GSE84796, and GSE57338) analyzed in this study can be found in NCBI Gene Expression Omnibus (GEO).

## Abbreviations

SARS-CoV-2: Severe acute respiratory syndrome coronavirus 2
COVID-19: Coronavirus disease 2019
hiPSC-CMs: Human induced pluripotent stem cell-derived cardiomyocytes
DEGs: Differentially expressed genes
ACE2: Angiotensin-converting enzyme 2
FC: Fold change
GO: Gene Ontology
KEGG: Kyoto Encyclopedia of Genes and Genomes
PPI: Protein–protein interaction
NCBI: National Center for Biotechnology Information
GEO: Gene Expression Omnibus
ISO: Isoproterenol
TAC: Transverse aortic constriction
FS: Fraction shortening
EF: Ejection fraction
qPCR: Quantitative real-time polymerase chain reaction
GAPDH: Glyceraldehyde-3-phosphate dehydrogenase
BP: Biological process
CC: Cellular component
MF: Molecular function
IL10RA: Interleukin 10 receptor subunit alpha
TLR7: Toll like receptor 7
CCR5: C–C motif chemokine receptor 5
CSF1R: Colony stimulating factor 1 receptor
SIGLEC1: Sialic acid binding Ig like lectin 1
CLEC10A: C-type lectin domain containing 10A
MRC1: Mannose receptor C-type 1
VWF: Von willebrand factor
PTPRC: Protein tyrosine phosphatase receptor type C
ITGAX: Integrin subunit alpha X
GZMB: Granzyme B
CCR7: C–C motif chemokine receptor 7
ITGAL: Integrin subunit alpha L
IL2RB: Interleukin 2 receptor subunit beta
ANP: Atrial natriuretic peptide
β-MHC: β-Myosin heavy chain
