# Supplementary material for: Landscape of molecular crosstalk between SARS-CoV-2 infection and cardiovascular diseases: emphasis on mitochondrial dysfunction and immune-inflammation
Source: J Transl Med. 2023 Dec 16;21:915. doi: 10.1186/s12967-023-04787-z (PMC10725609; doi:10.1186/s12967-023-04787-z)
Supplement: Supplementary file 1 — Additional file 1: Figure S1. Expression of host proteins predicted to interact with SARS-CoV-2 Spike protein based on structural analysis in the cardiovascular diseases-related GSE84796 dataset. Figure S2. Effect of isoproterenol (ISO) on H9c2 cells. Figure S3. Experimental verification of hub genes expression in isoproterenol induced hiPSC-CMs. Figure S4. Expression of cardiac hypertrophy and fibrosis markers in heart tissues of TAC mice. Table S1. Characteristics of heart tissue samples from heart failure dataset used in this study. Table S2. Primers used in this study. Table S3. Parameters of cardiac functions in two groups of mice. [file 12967_2023_4787_MOESM1_ESM.docx]

**Additional file**

**Landscape of molecular crosstalk between SARS-CoV-2 infection and cardiovascular diseases:** **emphasis on** **mitochondrial dysfunction and immune-inflammation**

Shiyu Dai^†^, Ting Cao^†^, Han Shen^†^, Xuejing Zong, Wenyu Gu, Hanghang Li, Lei Wei, Haoyue Huang, Yunsheng Yu, Yihuan Chen, Wenxue Ye, Fei Hua, Hongyou Fan, Zhenya Shen^*^

Department of Cardiovascular Surgery of the First Affiliated Hospital & Institute for Cardiovascular Science, Suzhou Medical College, Soochow University, Suzhou 215006, China

^*^Corresponding author

Correspondence should be address to Dr. Zhenya Shen, Department of Cardiovascular Surgery of the First Affiliated Hospital & Institute for Cardiovascular Science, Suzhou Medical College, Soochow University, Suzhou 215006, P.R. China; E-mail: uuzyshen@aliyun.com

Shiyu Dai, Ting Cao, and Han Shen equally contributed to this work.

**This file includes:**

Additional file 1: methods;

Additional file 1: Figures 4;

Additional file 1: Tables 3.

**Additional file 1: methods**

**Cell viability assay**

To examine the cytotoxic effect of isoproterenol (ISO) on H9c2 cardiomyocytes, Cell Counting Kit-8 reagent (CCK8, Beyotime, Cat#C0038) assay was performed. Briefly, H9c2 cells were seeded in 96-well plates at a density of 1.5 × 10^4^ cells per well and incubated with medium containing different concentrations of ISO (MCE, Cat#HY-B0468). After 24 hours of drug treatment, 10 μL of CCK8 reagent (in 100 μL of medium/well) was added and incubated with the cells for 1 hour. The absorbance at 450 nm was measured using a Biotek Synergy H1 microplate reader (Biotek). Cell viability of ISO-treated cells was normalized by comparing with cells without drug treatment.

**Cell culture and drug treatment**

Human inducible pluripotent stem-derived cardiomyocytes (hiPSC-CMs) were obtained from Cellapy (Cat#CA2201106) and maintained with Cardioeasy medium (Cellapy, Cat#CA2015002) at 37°C and 5% (v/v) CO2. hiPSC-CMs were treated with 1 μM β-adrenergic agonist ISO, which was reported to exert proarrhythmic effects on hiPSC-CMs [1-3], or vehicle (DMSO) for 24 hours. Total RNA was extracted with TRIzol reagent (Invitrogen, Cat#15596018) from the cells and relative gene expression levels were measured by qPCR (See also the Methods for more details).

**SARS-CoV-2 entry-associated receptors or co-factors**

Based on the literature reports, 17 receptors or co-factors associated with SARS-CoV-2 invasion, as well as host proteins predicted to interact with the Spike protein based on structural analysis were analyzed, as listed below:

**ACE2:** Angiotensin-converting enzyme 2 (ACE2) is the predominant receptor for SARS-CoV-2 infection and entry into cells [4, 5] (See also Background).

**TMPRSS2:** Transmembrane protease serine 2 (TMPRSS2) cleaves the Spike protein, which is a necessary step for fusion of virus to cellular membrane and entry cell [6].

**SIGLEC1:** Sialic acid binding Ig like lectin 1 (SIGLEC1), a lectin receptor, is reported to mediate the attachment of SARS-CoV-2 to antigen-presenting cells [7].

**CLEC10A:** [C-type lectin domain containing 10A](https://www.ncbi.nlm.nih.gov/gene/10462) (CLEC10A) interacts with the SARS-CoV-2 Spike protein through a region other than the receptor-binding structural domain, and this exposure to the virus induces a strong pro-inflammatory response in myeloid cells that correlates with the severity of COVID-19 [8].

**MRC1:** [Mannose receptor C-type 1](https://www.ncbi.nlm.nih.gov/gene/4360) (MRC1), also known as CD206, is a mannose receptor that is highly expressed in dendritic cells, monocytes, and macrophages and exhibits strong affinity to SARS-CoV-2 Spike protein [9].

**CD147:** CD147, also known as basigin (encoded by BSG), facilitates viral entry into host cells via endocytosis, and the interaction between CD147 and the Spike protein presents a noteworthy target for designing pharmaceuticals to combat COVID-19 [10, 11].

**VWF**: Von willebrand factor (vWF) is a marker of vascular endothelial cell phenotype. vWF may play a role in SARS-CoV-2 infected endothelial cells by regulating ACE2 expression [12].

**NRP1:** Neuropilin 1 (NRP1) binds to the C-end rule motif of S1 subunit of SARS-CoV-2 Spike protein to facilitate viral entry and infection [13]

**ASGR1 and KREMEN1:** Asialoglycoprotein receptor 1 (ASGR1) or kringle containing transmembrane protein 1 (KREMEN1) interact with SARS-CoV-2 Spike protein and play an important role in ACE2-independent viral entry [14].

**CD209 and CD209L:** CD209 (also known as DC-SIGN) and CD209L (also known as L-SIGN) belong to the C-type lectin superfamily and act as alternative receptors for SARS-CoV-2 in disease-relevant cell type, including the vascular system [15].

**AXL:** Tyrosine-protein kinase receptor (AXL) interacts with N-terminal domain of SARS-CoV-2 Spike protein and acts as a novel receptor for SARS-CoV-2 [16, 17].

**SR-B1:** The high-density lipoprotein (HDL) scavenger receptor B type 1 (SR-B1) facilitates ACE2-dependent entry of SARS-CoV-2 [18].

**DPP4:** Dipeptidyl peptidase 4/CD26 (DPP4/CD26) is a main cellular receptor for Middle East respiratory syndrome-related coronavirus (MERS-CoV). DPP4 is recognized as a potential receptor for SARS-CoV-2 because structural prediction shows that it has a direct interaction with the S1 domain of SARS-CoV-2 Spike protein [6, 11, 19].

**NLN and THOP1:** Neurolysin (NLN) and thimet oligopeptidase (THOP1) are closely-related zinc metallopeptidases. A study based on a computational approach predicting the interaction of Spike protein and ACE2-related proteins found that Spike protein may interact with NLN and THOP1, which have high structural similarity to ACE2 and have mitochondrial localization [20].

**Statistical analysis**

Statistical analysis was performed using GraphPad Prism 6.0. The statistical significance of differences between two groups was determined using Student’s *t*-test. Results were considered statistically significant at * *P* < 0.05, ** *P* < 0.01, *** *P* < 0.001, and **** *P* < 0.0001.

**Additional file 1: Figures**

**
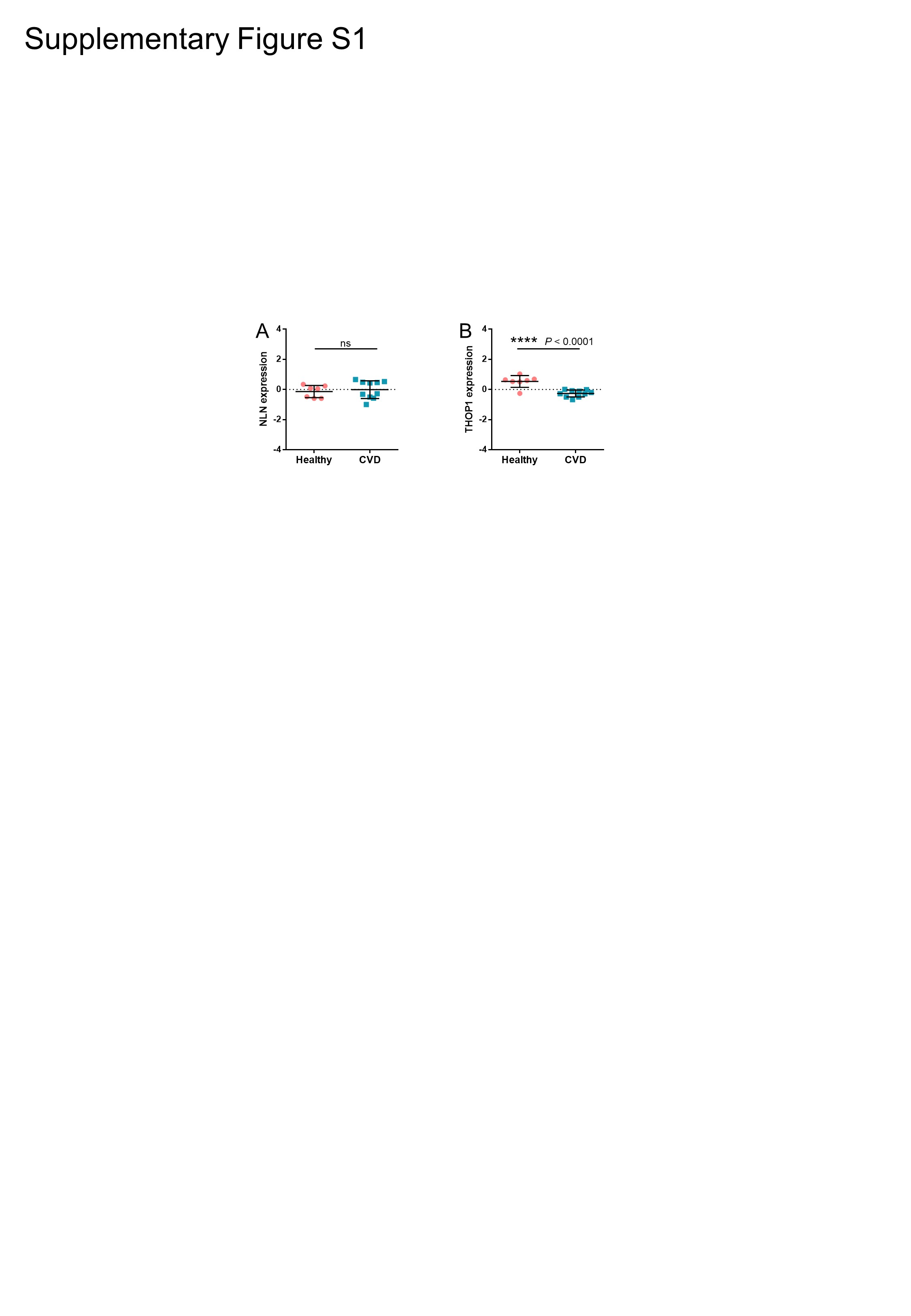
**

**Additional file 1: Figure. S1:** (A-B) Expression of host proteins predicted to interact with SARS-CoV-2 Spike protein based on structural analysis in the cardiovascular diseases-related GSE84796 dataset. CVD, cardiovascular disease. ****, *P* < 0.0001; ns, no significance.


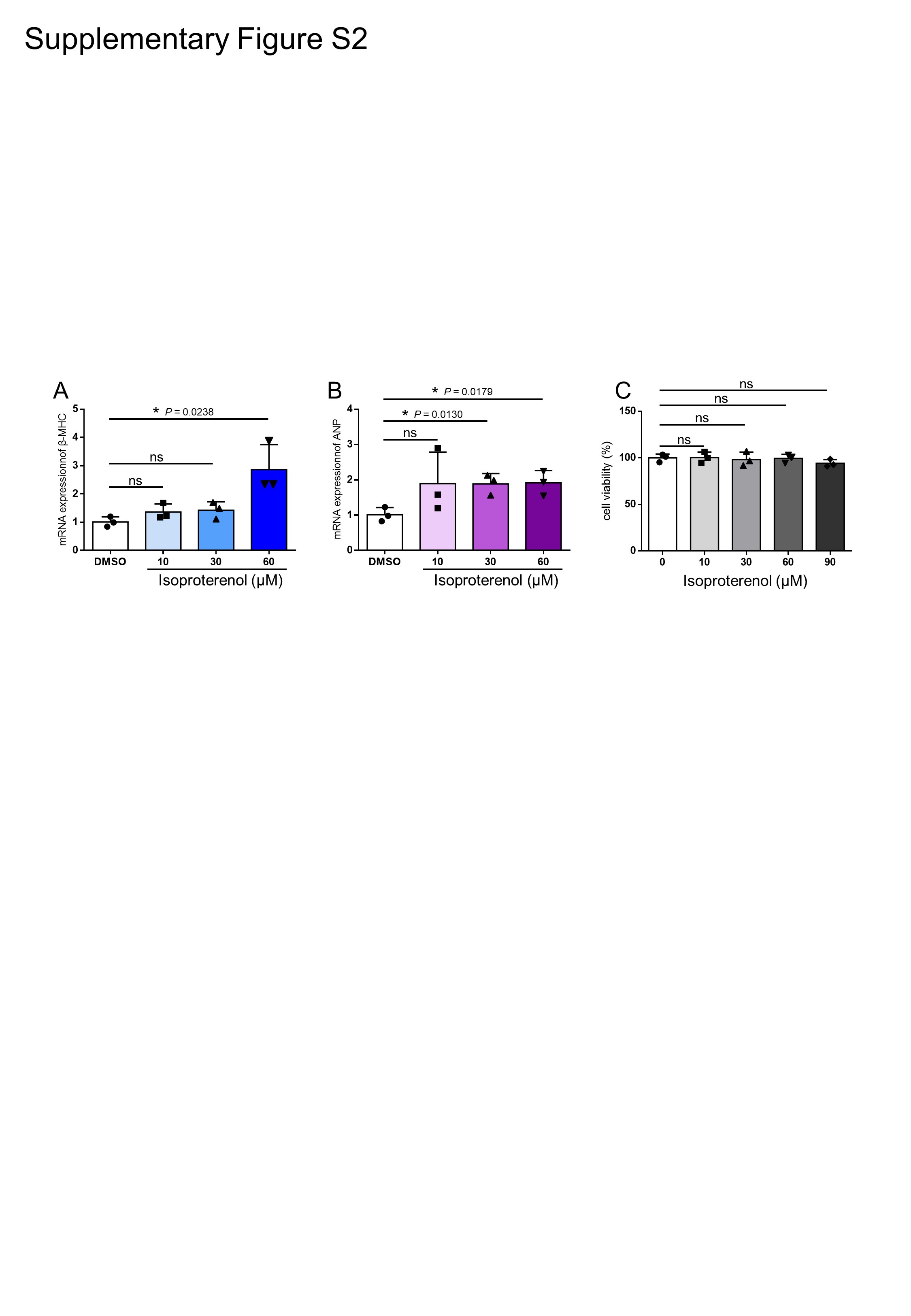


**Additional file 1: Figure. S2: Effect of isoproterenol (ISO) on H9c2 cells.** H9c2 cells were treated with indicated concentrations of ISO or vehicle (DMSO) for 24 hours. (A-B) Expression of mRNA expression of cardiac hypertrophy markers (ANP and β-MHC) were measured using qPCR (normalization was performed on Gapdh). (C) Cell viability was assessed using the CCK8 assay. Relative cell viability rates were calculated by normalization to the vehicle groups. Values represent means ± SD (n = 3 biologically independent samples). ANP, atrial natriuretic peptide; β-MHC, β-myosin heavy chain; Gapdh, Glyceraldehyde-3-phosphate dehydrogenase. *, *P* < 0.05; ns, not significant.


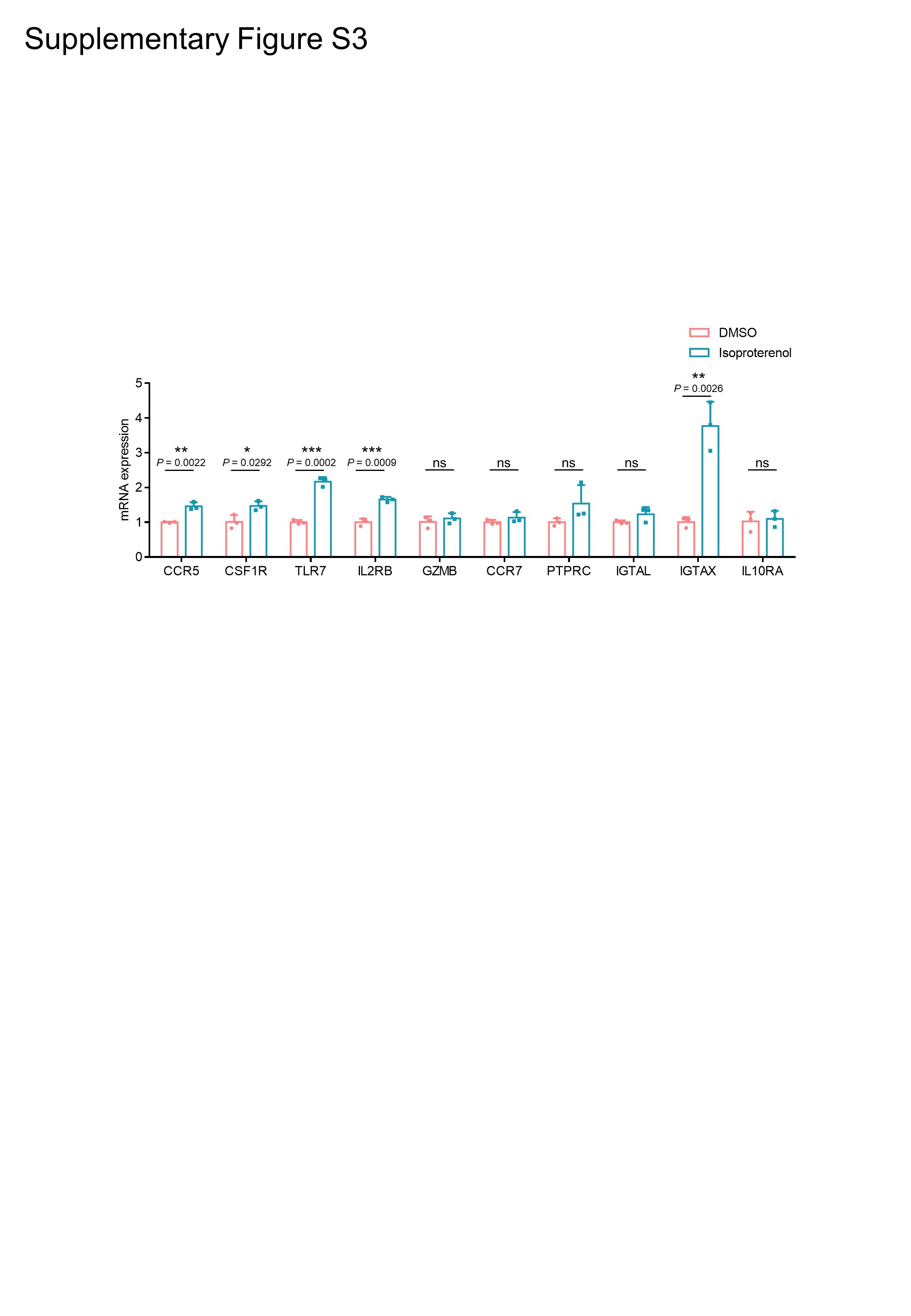


**Additional file 1: Figure. S3: Experimental verification of hub genes expression in isoproterenol induced hiPSC-CMs.** hiPSC-CMs treated with 1 μM ISO or vehicle (DMSO) for 24 h were performed to analyze mRNA expression levels of hub genes using qPCR (normalized to β-Actin). Values represent means ± SD (n = 3 biologically independent samples). *, *P* < 0.05; **, *P* < 0.01; ***, *P* < 0.001; ns, not significant.


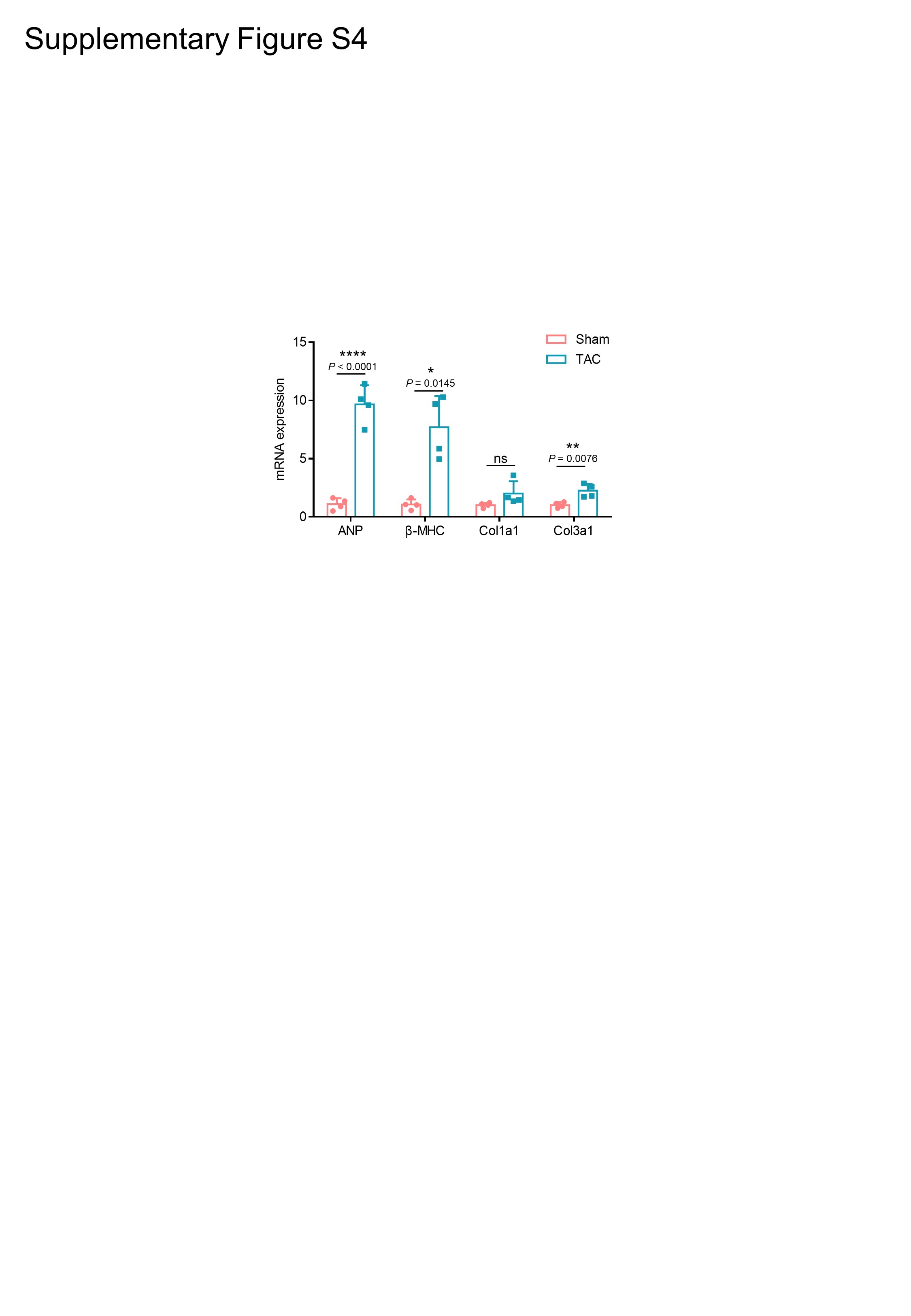


**Additional file 1: Figure. S4: Expression of cardiac hypertrophy and fibrosis markers in heart tissues of TAC mice.** Expression of mRNA levels of cardiac hypertrophy markers (ANP and β-MHC) and fibrosis markers (Col1a1 and Col3a1) in heart tissues were measured using qPCR (normalization was performed on Gapdh). Values represent means ± SD (n = 4 mice). ANP, atrial natriuretic peptide; β-MHC, β-myosin heavy chain; Col1a1, collagen type I alpha 1 chain; Col3a1, collagen type III alpha 1 chain; Gapdh, Glyceraldehyde-3-phosphate dehydrogenase. *, *P* < 0.05; **, *P* < 0.01; ****, *P* < 0.0001; ns, not significant.

**Additional file 1: Table S1.** **Characteristics of heart tissue samples from heart failure dataset used in this study.**

| **GEO accession** | **Group** | **Age** | **Sex** | **EF(%)** |
| --- | --- | --- | --- | --- |
| [GSM2251530](https://www.ncbi.nlm.nih.gov/geo/query/acc.cgi?acc=GSM2251530) | End-stage heart failure patients | 60 | Female | 20 |
| [GSM2251531](https://www.ncbi.nlm.nih.gov/geo/query/acc.cgi?acc=GSM2251531) | End-stage heart failure patients | 36 | Male | 25 |
| [GSM2251532](https://www.ncbi.nlm.nih.gov/geo/query/acc.cgi?acc=GSM2251532) | End-stage heart failure patients | 50 | Female | 23 |
| [GSM2251533](https://www.ncbi.nlm.nih.gov/geo/query/acc.cgi?acc=GSM2251533) | End-stage heart failure patients | 49 | Female | 15 |
| [GSM2251534](https://www.ncbi.nlm.nih.gov/geo/query/acc.cgi?acc=GSM2251534) | End-stage heart failure patients | 57 | Male | 29 |
| [GSM2251535](https://www.ncbi.nlm.nih.gov/geo/query/acc.cgi?acc=GSM2251535) | End-stage heart failure patients | 60 | Male | 64 |
| [GSM2251536](https://www.ncbi.nlm.nih.gov/geo/query/acc.cgi?acc=GSM2251536) | End-stage heart failure patients | 32 | Male | 12 |
| [GSM2251537](https://www.ncbi.nlm.nih.gov/geo/query/acc.cgi?acc=GSM2251536) | End-stage heart failure patients | 45 | Female | 20 |
| [GSM2251538](https://www.ncbi.nlm.nih.gov/geo/query/acc.cgi?acc=GSM2251536) | End-stage heart failure patients | 59 | Male | 17 |
| [GSM2251539](https://www.ncbi.nlm.nih.gov/geo/query/acc.cgi?acc=GSM2251536) | End-stage heart failure patients | 54 | Female | 36 |
| [GSM2251540](https://www.ncbi.nlm.nih.gov/geo/query/acc.cgi?acc=GSM2251536) | Healthy hearts of organ donors | -- | Male | -- |
| [GSM2251541](https://www.ncbi.nlm.nih.gov/geo/query/acc.cgi?acc=GSM2251536) | Healthy hearts of organ donors | 25 | Male | -- |
| [GSM2251542](https://www.ncbi.nlm.nih.gov/geo/query/acc.cgi?acc=GSM2251536) | Healthy hearts of organ donors | 22 | Male | -- |
| [GSM2251543](https://www.ncbi.nlm.nih.gov/geo/query/acc.cgi?acc=GSM2251536) | Healthy hearts of organ donors | 28 | Male | -- |
| [GSM2251544](https://www.ncbi.nlm.nih.gov/geo/query/acc.cgi?acc=GSM2251536) | Healthy hearts of organ donors | 46 | Male | -- |
| [GSM2251545](https://www.ncbi.nlm.nih.gov/geo/query/acc.cgi?acc=GSM2251536) | Healthy hearts of organ donors | 17 | Male | -- |
| [GSM2251546](https://www.ncbi.nlm.nih.gov/geo/query/acc.cgi?acc=GSM2251536) | Healthy hearts of organ donors | -- | Male | -- |

The data of heart failure patients used for analysis in this study was sourced from the GSE84796 dataset, which includes transcriptome analysis of human left ventricular free wall samples obtained from patients with end-stage heart failure at the time of heart transplantation (n = 10) and organ donors having no suitable recipient (n = 7) [21]. EF, ejection fraction.

**Additional file 1: Table S2. Primers used in this study.**

| **Primers** | **Forward (5’ to 3’)** | **Reverse (5’ to 3’)** |
| --- | --- | --- |
| Rat-ANP | AAAGCAAACTGAGGGCTCTGCTCG | TTCGGTACCGGAAGCTGTTGCA |
| Rat-β-MHC | TCTGGACAGCTCCCCATTCT | CAAGGCTAACCTGGAGAAGATG |
| Rat-Il2rb | GGTTACTTCTTCTTCCATT | AGTAGTCATCCTGTTCTC |
| Rat-Ccr7 | TTCAACATCACCAATAGC | GATGAAGGCATACAAGAA |
| Rat-Ccr5 | TATCTACCTGTTCAACCT | TAGCCAAGTACCTATCAA |
| Rat-Csf1r | CATTATCCACAAGGCTAA | AGTAGGTTCCAATATGATG |
| Rat-Gapdh | GACATGCCGCCTGGAGAA AC | AGCCCAGGATGCCCTTTAGT |
| Mouse-Il2rb | TGGAGCCTGTCCCTCTACG | TCCACATGCAAGAGACATTGG |
| Mouse-Gzmb | CCACTCTCGACCCTACATGG | GGCCCCCAAAGTGACATTTATT |
| Mouse-Tlr7 | ATGTGGACACGGAAGAGACAA | GGTAAGGGTAAGATTGGTGGTG |
| Mouse-Ptprc | ATGGTCCTCTGAATAAAGCCCA | TCAGCACTATTGGTAGGCTCC |
| Mouse-Ccr7 | TGTACGAGTCGGTGTGCTTC | GGTAGGTATCCGTCATGGTCTTG |
| Mouse-Itgax | CTGGATAGCCTTTCTTCTGCTG | GCACACTGTGTCCGAACTCA |
| Mouse-Il10ra | CCCATTCCTCGTCACGATCTC | TCAGACTGGTTTGGGATAGGTTT |
| Mouse-Ccr5 | TTTTCAAGGGTCAGTTCCGAC | GGAAGACCATCATGTTACCCAC |
| Mouse- Itgal | CCAGACTTTTGCTACTGGGAC | GCTTGTTCGGCAGTGATAGAG |
| Mouse-Csf1r | TGTCATCGAGCCTAGTGGC | CGGGAGATTCAGGGTCCAAG |
| Mouse-ANP | CTGCTAGACCACCTGGAGGA | AAGCTGTTGCAGCCTAGTCC |
| Mouse-β-MHC | TGCAAAGGCTCCAGGTCTGAGGGC | GCCAACACCAACCTGTCCAAGTTC |
| Mouse-Col1a1 | ACGGCTGCACGAGTCACAC | GGCAGGCGGGAGGTCTT |
| Mouse-Col3a1 | GTTCTAGAGGATGGCTGTACTAAACACA | TTGCCTTGCGTGTTTGATATTC |
| Mouse-Gapdh | TCTGGAAAGCTGTGGCGTG | CCAGTGAGCTTCCCGTTCAG |
| Homo-IL2RB | CAGCGGTGAATGGCACTTC | GGCATGGACTTGGCAGGAA |
| Homo-GZMB | CCCTGGGAAAACACTCACACA | GCACAACTCAATGGTACTGTCG |
| Homo-TLR7 | GCACAACTCAATGGTACTGTCG | GCACAACTCAATGGTACTGTCG |
| Homo-PTPRC | ACCACAAGTTTACTAACGCAAGT | TTTGAGGGGGATTCCAGGTAAT |
| Homo-CCR7 | TTTGAGGGGGATTCCAGGTAAT | GTAGGCCCACGAAACAAATGAT |
| Homo-ITGAX | GTAGGCCCACGAAACAAATGAT | AATTCCTCGAAAGTGAAGTGTGT |
| Homo-IL10RA | CCTCCGTCTGTGTGGTTTGAA | CACTGCGGTAAGGTCATAGGA |
| Homo-CCR5 | TTCTGGGCTCCCTACAACATT | TTGGTCCAACCTGTTAGAGCTA |
| Homo-ITGAL | TGCTTATCATCATCACGGATGG | CTCTCCTTGGTCTGAAAATGCT |
| Homo-CSF1R | GGGAATCCCAGTGATAGAGCC | TTGGAAGGTAGCGTTGTTGGT |
| Homo-ACTIN | CATCCGTAAAGACCTCTATGCCAAC | ATGGAGCCACCGATCCACA |

**Additional file 1: Table S3. Parameters of cardiac functions in two groups of mice.**

|  | **Sham** | **TAC** |
| --- | --- | --- |
| Heart rate (bmp) | 422.00 ± 12.14 | 433.25 ± 27.99 |
| EF (%) | 75.66 ± 1.51 | 47.29 ± 10.80** |
| FS (%) | 43.67 ± 1.55 | 23.79 ± 5.99** |
| LVAW;d (mm) | 0.64 ± 0.04 | 0.73 ± 0.12 |
| LVAW;s (mm) | 1.12 ± 0.13 | 1.18 ± 0.11 |
| LVID;d (mm) | 3.72 ± 0.36 | 4.39 ± 0.65 |
| LVID;s (mm) | 2.09 ± 0.18 | 3.38 ± 0.79* |
| LVPW;d (mm) | 0.81 ± 0.09 | 0.86 ± 0.14 |
| LVPW;s (mm) | 1.47 ± 0.17 | 1.14 ± 0.10* |
| LV Vol;d (μL) | 59.55 ± 13.00 | 89.93 ± 32.81 |
| LV Vol;s (μL) | 14.42 ± 2.98 | 50.91 ± 30.48 |

Values represent means ± SD (n = 4 mice). *, *P* < 0.05, **, *P* < 0.01 vs. sham group.

TAC, transverse aortic constriction; EF, ejection fraction; FS, fractional shortening; LVAW;d, left ventricular anterior wall dimension at end-diastole; LVAW;s, left ventricular anterior wall dimension at end-systole; LVID;d, left ventricle end diastolic inner diameter; LVID;s, left ventricle end systolic inner diameter; LVPW;d, left ventricle posterior wall thickness at end-diastole; LVPW;s, left ventricle posterior wall thickness at end-systole; LV Vol;d, left ventricular diastolic volume; LV Vol;s, left ventricular systolic volume.

**Reference**

1. Knollmann BC: **Induced pluripotent stem cell-derived cardiomyocytes: boutique science or valuable arrhythmia model?** *Circ Res* 2013, **112:**969-976; discussion 976.

2. Smith AS, Macadangdang J, Leung W, Laflamme MA, Kim DH: **Human iPSC-derived cardiomyocytes and tissue engineering strategies for disease modeling and drug screening.** *Biotechnol Adv* 2017, **35:**77-94.

3. Bezzerides VJ, Caballero A, Wang S, Ai Y, Hylind RJ, Lu F, Heims-Waldron DA, Chambers KD, Zhang D, Abrams DJ, Pu WT: **Gene Therapy for Catecholaminergic Polymorphic Ventricular Tachycardia by Inhibition of Ca(2+)/Calmodulin-Dependent Kinase II.** *Circulation* 2019, **140:**405-419.

4. Bian J, Li Z: **Angiotensin-converting enzyme 2 (ACE2): SARS-CoV-2 receptor and RAS modulator.** *Acta Pharm Sin B* 2021, **11:**1-12.

5. Bourgonje AR, Abdulle AE, Timens W, Hillebrands JL, Navis GJ, Gordijn SJ, Bolling MC, Dijkstra G, Voors AA, Osterhaus AD, et al: **Angiotensin-converting enzyme 2 (ACE2), SARS-CoV-2 and the pathophysiology of coronavirus disease 2019 (COVID-19).** *J Pathol* 2020, **251:**228-248.

6. Singh M, Bansal V, Feschotte C: **A Single-Cell RNA Expression Map of Human Coronavirus Entry Factors.** *Cell Rep* 2020, **32:**108175.

7. Perez-Zsolt D, Munoz-Basagoiti J, Rodon J, Elosua-Bayes M, Raich-Regue D, Risco C, Sachse M, Pino M, Gumber S, Paiardini M, et al: **SARS-CoV-2 interaction with Siglec-1 mediates trans-infection by dendritic cells.** *Cell Mol Immunol* 2021, **18:**2676-2678.

8. Lu Q, Liu J, Zhao S, Gomez Castro MF, Laurent-Rolle M, Dong J, Ran X, Damani-Yokota P, Tang H, Karakousi T, et al: **SARS-CoV-2 exacerbates proinflammatory responses in myeloid cells through C-type lectin receptors and Tweety family member 2.** *Immunity* 2021, **54:**1304-1319 e1309.

9. Gao C, Zeng J, Jia N, Stavenhagen K, Matsumoto Y, Zhang H, Li J, Hume AJ, Muhlberger E, van Die I, et al: **SARS-CoV-2 Spike Protein Interacts with Multiple Innate Immune Receptors.** *bioRxiv* 2020.

10. Wang K, Chen W, Zhang Z, Deng Y, Lian JQ, Du P, Wei D, Zhang Y, Sun XX, Gong L, et al: **CD147-spike protein is a novel route for SARS-CoV-2 infection to host cells.** *Signal Transduct Target Ther* 2020, **5:**283.

11. Radzikowska U, Ding M, Tan G, Zhakparov D, Peng Y, Wawrzyniak P, Wang M, Li S, Morita H, Altunbulakli C, et al: **Distribution of ACE2, CD147, CD26, and other SARS-CoV-2 associated molecules in tissues and immune cells in health and in asthma, COPD, obesity, hypertension, and COVID-19 risk factors.** *Allergy* 2020, **75:**2829-2845.

12. Furini G, De Carli A, Fonnesu R, Spezia PG, Scebba F, Pistello M, Lai M, Lionetti V: **Gene silencing of endothelial von Willebrand factor reduces the susceptibility of human endothelial cells to SARS-CoV-2 infection.** *FEBS J* 2023, **290:**4300-4315.

13. Daly JL, Simonetti B, Klein K, Chen KE, Williamson MK, Anton-Plagaro C, Shoemark DK, Simon-Gracia L, Bauer M, Hollandi R, et al: **Neuropilin-1 is a host factor for SARS-CoV-2 infection.** *Science* 2020, **370:**861-865.

14. Gu Y, Cao J, Zhang X, Gao H, Wang Y, Wang J, He J, Jiang X, Zhang J, Shen G, et al: **Receptome profiling identifies KREMEN1 and ASGR1 as alternative functional receptors of SARS-CoV-2.** *Cell Res* 2022, **32:**24-37.

15. Amraei R, Yin W, Napoleon MA, Suder EL, Berrigan J, Zhao Q, Olejnik J, Chandler KB, Xia C, Feldman J, et al: **CD209L/L-SIGN and CD209/DC-SIGN Act as Receptors for SARS-CoV-2.** *ACS Cent Sci* 2021, **7:**1156-1165.

16. Zeng C, Ye Z, Fu L, Ye Y: **Prediction analysis of porcine AXL protein as a potential receptor for SARS-CoV-2.** *J Infect* 2022, **84:**579-613.

17. Wang S, Qiu Z, Hou Y, Deng X, Xu W, Zheng T, Wu P, Xie S, Bian W, Zhang C, et al: **AXL is a candidate receptor for SARS-CoV-2 that promotes infection of pulmonary and bronchial epithelial cells.** *Cell Res* 2021, **31:**126-140.

18. Wei C, Wan L, Yan Q, Wang X, Zhang J, Yang X, Zhang Y, Fan C, Li D, Deng Y, et al: **HDL-scavenger receptor B type 1 facilitates SARS-CoV-2 entry.** *Nat Metab* 2020, **2:**1391-1400.

19. Vankadari N, Wilce JA: **Emerging WuHan (COVID-19) coronavirus: glycan shield and structure prediction of spike glycoprotein and its interaction with human CD26.** *Emerg Microbes Infect* 2020, **9:**601-604.

20. Tragni V, Preziusi F, Laera L, Onofrio A, Mercurio I, Todisco S, Volpicella M, De Grassi A, Pierri CL: **Modeling SARS-CoV-2 spike/ACE2 protein-protein interactions for predicting the binding affinity of new spike variants for ACE2, and novel ACE2 structurally related human protein targets, for COVID-19 handling in the 3PM context.** *EPMA J* 2022, **13:**149-175.

21. Laugier L, Frade AF, Ferreira FM, Baron MA, Teixeira PC, Cabantous S, Ferreira LRP, Louis L, Rigaud VOC, Gaiotto FA, et al: **Whole-Genome Cardiac DNA Methylation Fingerprint and Gene Expression Analysis Provide New Insights in the Pathogenesis of Chronic Chagas Disease Cardiomyopathy.** *Clin Infect Dis* 2017, **65:**1103-1111.
